# Effectiveness of intraoperative peritoneal lavage with saline in patient with intra-abdominal infections: a systematic review and meta-analysis

**DOI:** 10.1186/s13017-023-00496-6

**Published:** 2023-03-29

**Authors:** Qi Zhou, Wenbo Meng, Yanhan Ren, Qinyuan Li, Marja A. Boermeester, Peter Muli Nthumba, Jennifer Rickard, Bobo Zheng, Hui Liu, Qianling Shi, Siya Zhao, Zijun Wang, Xiao Liu, Zhengxiu Luo, Kehu Yang, Yaolong Chen, Robert G. Sawyer

**Affiliations:** 1 Evidence-Based Medicine Center, School of Basic Medical Sciences, Lanzhou University, Lanzhou, China; 2 Department of General Surgery, The First Hospital of Lanzhou University, Lanzhou, China; 3 University of Massachusetts Chan Medical School, Worcester, MA USA; 4 Department of Respiratory Medicine, Children’s Hospital of Chongqing Medical University, National Clinical Research Center for Child Health and Disorders, Ministry of Education Key Laboratory of Child Development and Disorders, Chongqing Key Laboratory of Pediatrics, Chongqing, China; 5 Department of Surgery, Amsterdam UMC Location AMC, University of Amsterdam, Amsterdam, The Netherlands; 6 Amsterdam Gastroenterology Endocrinology and Metabolism, Amsterdam, The Netherlands; 7 Department of Plastic Surgery, AIC Kijabe Hospital, Kijabe, Kenya; 8 Vanderbilt University Medical Center, Nashville, TN USA; 9 Department of Surgery, University of Minnesota, Minneapolis, MN USA; 10 Department of Gastrointestinal Surgery, West China Hospital, Sichuan University, Chengdu, China; 11 School of Public Health, Lanzhou University, Lanzhou, China; 12 The First School of Clinical Medicine, Lanzhou University, Lanzhou, China; 13 Research Unit of Evidence-Based Evaluation and Guidelines, Chinese Academy of Medical Sciences (2021RU017), School of Basic Medical Sciences, Lanzhou University, Lanzhou, China; 14 Chevidence Lab of Child and Adolescent Health, Children’s Hospital of Chongqing Medical University, Chongqing, China; 15 Department of Surgery, Western Michigan University School of Medicine, Kalamazoo, MI USA

**Keywords:** Intraoperative peritoneal lavage, Intra-abdominal infection, Meta-analysis

## Abstract

**Background:**

Intraoperative peritoneal lavage (IOPL) with saline has been widely used in surgical practice. However, the effectiveness of IOPL with saline in patients with intra-abdominal infections (IAIs) remains controversial. This study aims to systematically review randomized controlled trials (RCTs) evaluating the effectiveness of IOPL in patients with IAIs.

**Methods:**

The databases of PubMed, Embase, Web of Science, Cochrane library, CNKI, WanFang, and CBM databases were searched from inception to December 31, 2022. Random-effects models were used to calculate the risk ratio (RR), mean difference, and standardized mean difference. The Grading of Recommendations Assessment, Development and Evaluation (GRADE) was used to rate the quality of the evidence.

**Results:**

Ten RCTs with 1318 participants were included, of which eight studies on appendicitis and two studies on peritonitis. Moderate-quality evidence showed that the use of IOPL with saline was not associated with a reduced risk of mortality (0% vs. 1.1%; RR, 0.31 [95% CI, 0.02–6.39]), intra-abdominal abscess (12.3% vs. 11.8%; RR, 1.02 [95% CI, 0.70–1.48]; *I*^*2*^ = 24%), incisional surgical site infections (3.3% vs. 3.8%; RR, 0.72 [95% CI, 0.18–2.86]; *I*^*2*^ = 50%), postoperative complication (11.0% vs. 13.2%; RR, 0.74 [95% CI, 0.39–1.41]; *I*^*2*^ = 64%), reoperation (2.9% vs. 1.7%; RR,1.71 [95% CI, 0.74–3.93]; *I*^*2*^ = 0%) and readmission (5.2% vs. 6.6%; RR, 0.95 [95% CI, 0.48–1.87]; *I*^*2*^ = 7%) in patients with appendicitis when compared to non-IOPL. Low-quality evidence showed that the use of IOPL with saline was not associated with a reduced risk of mortality (22.7% vs. 23.3%; RR, 0.97 [95% CI, 0.45–2.09], *I*^*2*^ = 0%) and intra-abdominal abscess (5.1% vs. 5.0%; RR, 1.05 [95% CI, 0.16–6.98], *I*^*2*^ = 0%) in patients with peritonitis when compared to non-IOPL.

**Conclusion:**

IOPL with saline use in patients with appendicitis was not associated with significantly decreased risk of mortality, intra-abdominal abscess, incisional surgical site infection, postoperative complication, reoperation, and readmission compared with non-IOPL. These findings do not support the routine use of IOPL with saline in patients with appendicitis. The benefits of IOPL for IAI caused by other types of abdominal infections need to be investigated.

**Supplementary Information:**

The online version contains supplementary material available at 10.1186/s13017-023-00496-6.

## Background

Intra-abdominal infections (IAIs) are common surgical emergencies and have become the second leading cause of sepsis in patients in the intensive care unit, following respiratory infection [1–3]. The results of a study showed that the mortality was 4.4% in complicated IAI with sepsis and 67.8% in complicated IAI with septic shock [4]. The mortality of IAI varied greatly due to different infection sites and pathogens, and the overall mortality caused by complicated IAIs was about 10% [5, 6]. Therefore, the World Society of Emergency Surgery (WSES), the World Surgical Infection Society (WSIS), the Infectious Diseases Society of America (IDSA), the Canadian Surgical Society (CSS), the Chinese Society of Surgical Infection and Intensive Care (CSSIIC) and other organizations have developed clinical practice guidelines (CPGs) to address serious harms caused by IAIs [7–11].

Intraoperative peritoneal lavage (IOPL) is a widely used approach to control the source of infection in patients with IAIs [12, 13]. However, the effectiveness of IOPL has been controversial since it was first proposed in 1905 [14]. A study showed that compared with no irrigation, the use of IOPL reduced the risk of intra-abdominal abscess (7.7% vs. 19.4%, *P* < 0.0001), but there was no significant difference in incisional surgical site infection (0.4% vs. 1.8%, P = 0.05) [15]. However, another retrospective study showed that compared with no irrigation, the use of IOPL increased the risk of intra-abdominal abscess (17.2% vs. 4.0%, *P* = 0.002) and incisional surgical site infection (SSI) (8.6% vs. 1.0%, *P* = 0.003) [16]. The recommendations on the use of IOPL in patients with IAIs vary greatly across current CPGs due to current contradictory evidence [7, 8]. Therefore, a systematic review (SR) is needed so that evidence-based recommendations can be formulated to guide the proper use of IOPL.

Several SRs aimed to investigate the effectiveness of IOPL, but these reviews only focused on appendicitis, ignoring other types of abdominal infections [17–22]. In addition, they mainly included observational studies, the quality of which was low or very low, for data synthesis [17–19, 22]. Furthermore, they did not analyze some important outcomes such as mortality, reoperation, and readmission and outcomes by the extent of scope of infection, the volume of irrigation, and the type of population [17–22].

Therefore, this SR aims to comprehensively explore the effectiveness of IOPL with saline in patients with IAI and to analyze whether the type of infection, the volume of flushing and the type of population affect the effectiveness of IOPL. The findings from our review can help clinicians in their daily practice and will inform future CPGs.

## Methods

This SR was performed in accordance with the *Cochrane Handbook* [23]. We report the results in accordance with the *Preferred Reporting Items for Systematic Reviews and Meta-Analysis* (PRISMA) statement [24]. This review has been registered on the PROSPERO (CRD42019145109) and the protocol has been published [25].

### Search strategy

We searched MEDLINE, the Cochrane Library, Web of Science, EMBASE, China National Knowledge Infrastructure (CNKI), WanFang, and China Biology Medicine disc (CBM) databases from the inception dates to December 31, 2022. We used database-specific combinations of the following search terms and phrases: *intra-abdominal infections, peritoneal sepsis, intraperitoneal infection, peritonitis, appendicitis, stomach rupture, irrigation, lavage, intraoperation, surgery,* and their derivatives. The details of search strategy are shown in Additional file 1: Table S1. Supplementary searches were conducted on Google and clinical trial registry platforms. Finally, we reviewed the references from the included articles manually to identify any missed potentially studies. The inclusion of studies was not restricted by publication status or language.

### Eligibility criteria

Trials were selected based on the following inclusion criteria: (1) patients diagnosed with IAIs and requiring surgery, regardless of age, gender and other factors; (2) all patients in the intervention group underwent IOPL with normal saline (Ringer's solution was regarded as normal saline) during operation, the control group were only treated with conventional aspiration; and (3) randomized controlled trials.

### Study selection

Four groups of investigators performed study selection independently. There were three stages of screening: (1) In phase one, we screened titles and abstracts of search results to exclude literature that obviously did not meet the inclusion and exclusion criteria; (2) In phase two, full-text articles were obtained for articles identified by one or both investigators as potentially relevant; (3) In phase three, the full texts of eligible articles were reviewed independently by the same two researchers. Any disagreements were solved through discussion or consultation with a third investigator.

### Data extraction

Two researchers independently extracted the following information from each study: (1) basic information: the first author, publication year, country, type of population, type of disease, scope of infection, age and gender, etc.; (2) intervention protocol: type of procedure, irrigation volume and follow-up, etc.; (3) outcome: the primary outcomes are mortality and intra-abdominal abscess (IAA). Detailed definition for each outcome was described in Additional file 1: Table S2. If sufficient data were not available, we contacted the authors of studies by email to request them or calculated from other reported data according to methods recommended by the Cochrane Handbook (Additional file 1: Table S3) [23].

### Risk of bias and quality of evidence

Two researchers independently assessed the risk of bias (RoB) of the included RCTs using the Cochrane RoB tool [23]. The RoB of each RCT was evaluated based on seven items: random sequence generation, allocation concealment, blinding of participants and personnel, blinding of outcome assessment, incomplete outcome data, selective reporting, and other bias. Each item was graded as low risk, high risk, or unclear risk. We resolved disagreements by discussion or by consultation with another investigator. We assessed the quality of the evidence with the Grading of Recommendations Assessment, Development and Evaluation (GRADE) approach for all outcomes [26]. The quality of meta-analysis of RCTs starts at high quality and can be downgraded based on risk of bias, indirectness, imprecision, inconsistency, and publication bias to levels of moderate, low, and very low quality. We performed the assessment using the GRADEpro software and generated a summary of findings table [27].

### Data analysis

We did our data analysis with RevMan 5.4 software and STATA15.0 (StataCorp, College Station, Texas, USA). We used a random-effects model and pooled risk ratios (RR) with 95% confidence intervals (CI) for dichotomous outcomes and mean differences (MD) or standardized mean difference (SMD) with 95% CI for continuous outcomes [28]. Heterogeneity was assessed by the* I*^2^ statistic and values of 25%, 50%, and 75% were considered low, moderate, and high, respectively [23, 29].

We performed pre-specified subgroup analysis on the following variables: (1) type of infection: patients are divided into diffuse (e.g., diffuse peritonitis) or localized (e.g., limited to a certain organ area, such as local infection of the appendix) IAIs based on the infection area involved; (2) type of population (child or adult): child was defined as younger than 18 years old, and adult was 18 years and older; (3) irrigation volume (≥ 3 or < 3 L): it is determined by the average or median flushing volume; (4) country income level (high-income (HIC) or low- and middle-income (LMIC)): according to the World Bank standard. Due to most of the articles did not clearly define the type of infection and population, we judged these based on inclusion criteria, baseline characteristics, and volume of peritoneal flushing. We also performed a sensitivity analysis to assess the robustness of our findings by excluding one research for every analysis [23]. Publication bias was detected by Egger’s test [30].

## Results

**Fig. 1:**
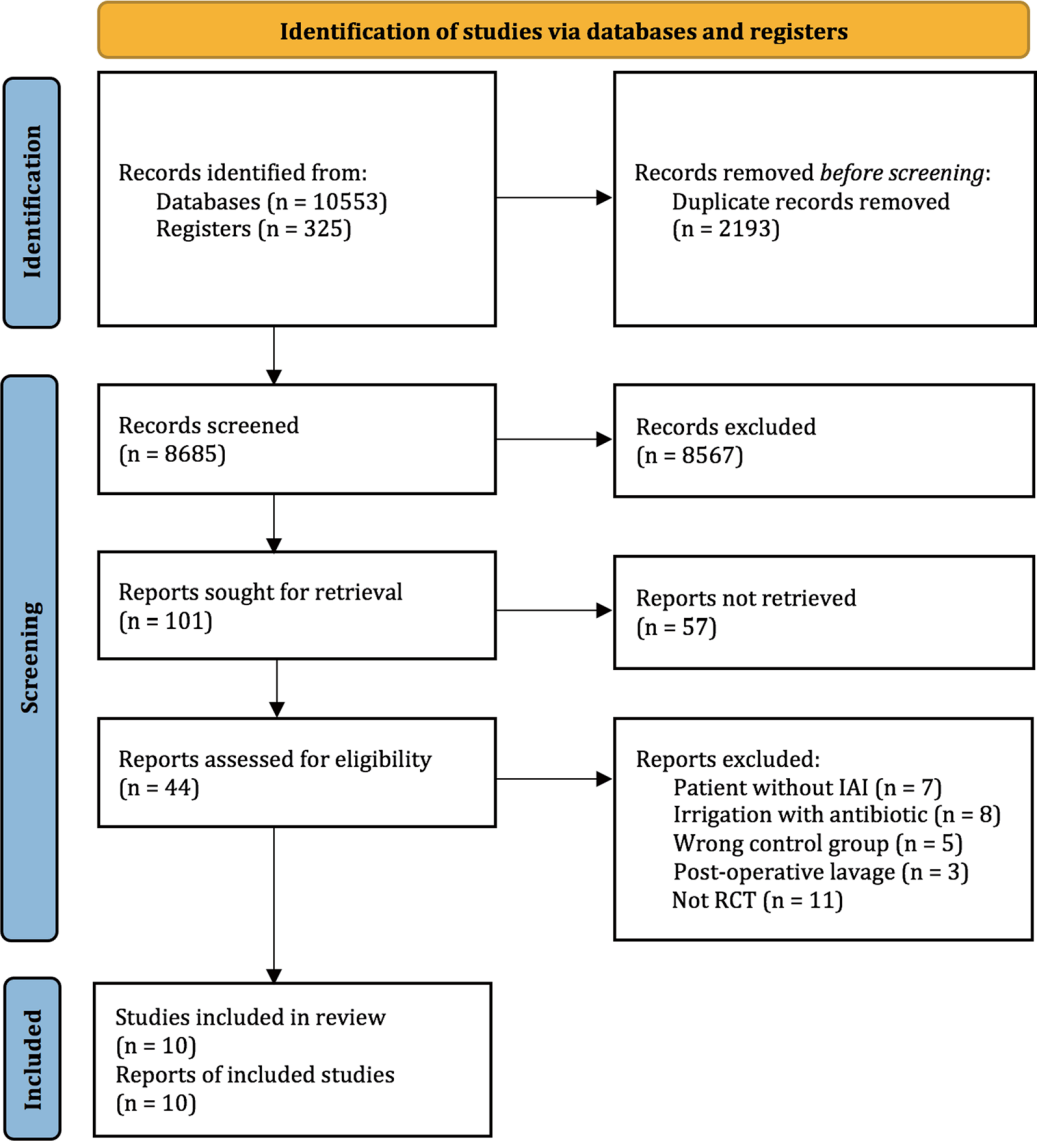
Literature search and screening process

Overall, the combined search identified 10,878 records, of which 10,834 were excluded based on duplicates and the title and abstract evaluation. The remaining 44 articles underwent full-text evaluation, and 34 were excluded (Additional file 1: Table S4). Finally, ten RCTs including 1318 patients were included [31–40]. The PRISMA diagram of the study selection process is shown in Fig. 1.

### Characteristics of included studies

Ten RCTs were published from 1982 to 2020, nine were journal papers [31–38, 40] and one was a doctoral thesis [39]. Eight RCTs [33–40] focused on appendicitis and two RCTs [31, 32] focused on peritonitis caused by perforation or injury to the stomach, duodenum, small intestine, appendix, etc. No studies focused on infectious pancreatitis or fecal peritonitis. Eight RCTs involved adults [31, 32, 34–37, 39, 40] and two involved children [33, 38]. The majority of the patients with peritonitis involved in two RCTs [31, 32] were diffuse infections, and the patients with appendicitis involved in eight RCTs were localized infections [33–40]. Peritonitis studies have mainly focused on the outcomes of mortality, intra-abdominal abscess (IAA), incisional surgical site infections (SSI), and postoperative complications. Studies on appendicitis not only examine these outcomes, but also evaluate reoperation, readmission, operative time, length of stay, and hospital charges. Eight RCTs performed laparoscopic appendectomy [33–40] and two RCTs performed open surgery [31, 32] (Table 1). The RoB assessment showed that none of the included RCTs were blinded to participants and personnel. All RCTs did not specify whether the assessment of the outcome was blinded, six RCTs were unclear about allocation concealment, and four RCTs were unclear about random sequence generation (Additional file 1: Fig. S1).

**Table 1:** Characteristics of the included studies

Study ID	Country	Type of disease^a^	Type of population	Type of procedure	Irrigation volume (L)^d^	Sample size	Age (year)^c^		Sex (male, %)		Preoperative antibiotic (%)		Postoperative antibiotic (%)		Follow-up (day)
							IOPL	Non-IOPL	IOPL	Non-IOPL	IOPL	Non-IOPL	IOPL	Non-IOPL	
Hunt [31]	USA	Peritonitis	Adult^b^	OS	4 (2–10)	29	50 (15–84)		NR	NR	100	100	100	100	< 30
Schein et al. [32]	South Africa	Peritonitis	Adult	OS	> 5	58	54 (21–91)	51 (18–90)	51.7	55.2	100	100	100	100	30
St Peter et al. [33]	USA	PA	Child	LA	0.9 (0.5–2)	220	10.4 ± 3.8	9.7 ± 3.6	52.7	59.1	100	100	100	100	28
Snow et al. [34]	Australia	PA	Adult	LA	0.7 (0.5–1.0)	81	31.1 ± 12.7	26.4 ± 13.8	67.5	61.0	100	100	52.5	41.5	42
Sun et al. [35]	China	CA	Adult	LA	3.1 ± 0.8	260	37.9 ± 19.1	38.7 ± 18.5	56.2	54.6	NR	NR	NR	NR	< 30
Wang et al. [36]	China	AP	Adult	LA	NR	78	35.9 ± 2.2	35.6 ± 2.1	53.8	51.3	NR	NR	100	100	< 30
Sardiwalla et al. [37]	South Africa	CA	Adult^b^	LA	3	86	25.7 ± 17.0	27.4 ± 11.9	45.2	65.9	NR	NR	100	100	42
Nataraja et al. [38]	Australia	CA	Child	LA	2.4 (2–5)	86	9.5 (3–16.0)	10 (4–16.0)	47.7	45.2	100	100	100	100	42
Palao et al. [39]	Spain	CA	Adult	LA	> 0.3	134	47.0 ± 18.0	43.0 ± 15.9	37.9	27.9	100	100	100	100	30
Gemici et al. [40]	Turkey	PA	Adult^b^	LA	0.5	286	36.2 ± 18.6	34.5 ± 17.4	65.2	65.5	NR	NR	100	100	< 30

*PA* perforated appendicitis; *CA* complicated appendicitis; *AP* acute appendicitis; *OS* open surgery; *LA*: laparoscopic appendectomy; *NR* not report; *IOPL* intraoperative peritoneal lavage with saline; *Non-IOPL* Intraoperative peritoneal lavage with saline was not performed

^a^Peritonitis: Peritoneal infection (almost diffuse infection) caused by perforation or injury to the stomach, duodenum, small intestine, appendix, etc. Patients with diffuse fecal peritonitis, infected pancreatic necrosis, or postoperative peritonitis were not included

^b^Almost all of the patients included in the study were adults

^c^Data are reported as mean ± SD or median (range)

### Mortality

**Fig. 2:**
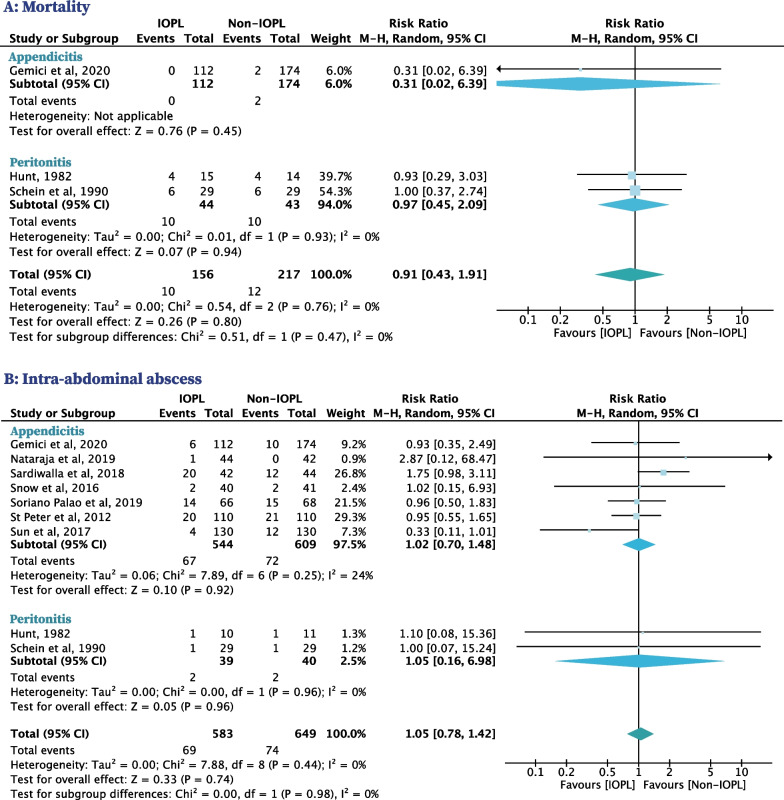
Primary outcomes in patients with IAIs who used IOPL compared with patients who did not

Three RCTs with 373 patients reported on mortality, one included patients with appendicitis and two included patients with peritonitis [31, 32, 40]. There were no reported deaths in the IOPL group and two (1.1%) in the non-IOPL group. The use of IOPL was not significantly associated with a decreased risk of mortality compared to non-IOPL for patients with appendicitis (RR, 0.31 [95% CI, 0.02–6.39]) (Fig. 2A). Ten patients with peritonitis (22.7%) died in the IOPL group, compared to 10 patients (23.3%) in the non-IOPL group. The use of IOPL was not significantly associated with a decreased risk of mortality compared to non-IOPL for patients with peritonitis (RR, 0.97 [95% CI, 0.45–2.09], *I*^*2*^ = 0%) (Fig. 2A). No significant differences in mortality were found in other subgroups stratified by the type of population (child: no data on mortality; adult: RR, 0.91 [95% CI, 0.43–1.91]), irrigation volume (< 3L: RR, 0.31 [95% CI, 0.02–6.39]; ≥ 3L: RR, 0.97 [95% CI, 0.45–2.09]), and country income level (HIC: RR, 0.93 [95% CI, 0.29–3.03]; LMIC: RR, 0.89 [95% CI, 0.34–2.31]) (Table 2, Additional file 1: Fig. S2).

**Table 2:** Subgroup analysis of primary outcomes in patients with IAIs who used IOPL compared with patients who did not

Variable	No. of trials	No. of participants		RR (95% CI)	*P* value^b^
		Events/total	Rate (%)		
*1. Mortality*					
Type of population					
Child	0	0/0	0	NA	NA
Adult	3	22/373	5.9	0.91 (0.43–1.91)	
Irrigation volume^a^					
< 3 L	1	2/286	0.7	0.31 (0.02, 6.39)	0.47
≥ 3 L	2	20/87	23.0	0.97 (0.45, 2.09)	
Country income level					
HIC	1	8/29	27.6	0.93 (0.29, 3.03)	0.95
LMIC	2	14/344	4.1	0.89 (0.34, 2.31)	
*2. Intra-abdominal abscess*					
Type of population					
Child	2	42/306	14.1	0.98 (0.57, 1.70)	0.92
Adult	7	101/926	10.9	1.02 (0.66, 1.58)	
Irrigation volume^a^					
< 3 L	5	91/807	12.0	0.97 (0.67, 1.41)	0.90
≥ 3 L	4	52/425	12.2	0.90 (0.30, 2.67)	
Country income level					
HIC	5	77/542	14.2	0.98 (0.66, 1.46)	0.88
LMIC	4	66/690	9.6	0.91 (0.39, 2.12)	

*NA* not estimable; *HIC* high-income; *LMIC* low- and middle-income

^a^The mean or medium irrigation volume

^b^P value for subgroup difference

### IAA

Nine RCTs with 1232 patients reported on IAA, seven included patients with appendicitis and two included patients with peritonitis [31–35, 37–40]. IAA occurred in 67 patients with appendicitis (12.3%) in the IOPL group and 72 patients with appendicitis (11.8%) in the non-IOPL group. The use of IOPL was not significantly associated with a decreased risk of IAA compared to non-IOPL for patients with appendicitis (RR, 1.02 [95% CI, 0.70–1.48], *I*^*2*^ = 24%) (Fig. 2B). IAA occurred in two patients with peritonitis (5.1%) in the IOPL group and two patients with peritonitis (5.0%) in the non-IOPL group. The use of IOPL was not significantly associated with a decreased risk of IAA compared to non-IOPL for patients with peritonitis (RR, 1.05 [95% CI, 0.16–6.98],* I*^2^ = 0%) (Fig. 2B). No significant differences in IAA were found in other subgroups stratified by the type of population (child: RR, 0.98 [95% CI, 0.57–1.70]; adult: RR, 1.02 [95% CI, 0.66–1.58]), irrigation volume (< 3L: RR, 0.97 [95% CI, 0.67–1.41]; ≥ 3L: RR, 0.90 [95% CI, 0.30–2.67]), and country income level (HIC: RR, 0.98 [95% CI, 0.66–1.46]; LMIC: RR, 0.91 [95% CI, 0.39–2.12]) (Table 2, Additional file 1: Fig. S2).

### Incisional SSI

**Fig. 3:**
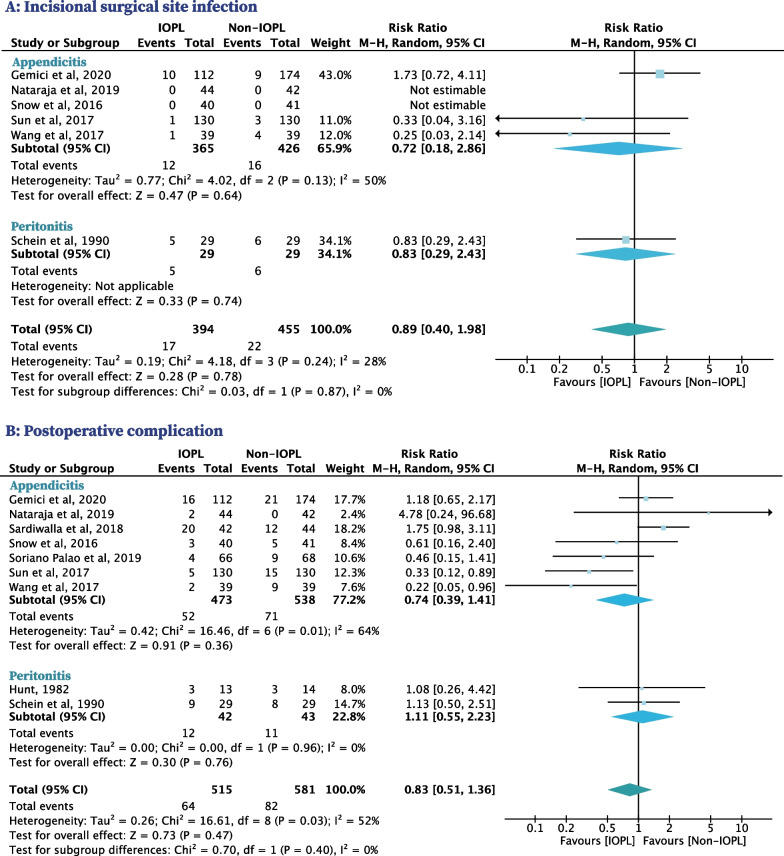
Secondary outcomes in patients with IAIs who used IOPL compared with patients who did not

Six RCTs with 849 patients reported on incisional SSI, five included patients with appendicitis and one included patients with peritonitis [32, 34–36, 38, 40]. Incisional SSI occurred in 12 patients with appendicitis (3.3%) in the IOPL group and 16 patients with appendicitis (3.8%) in the non-IOPL group. The use of IOPL was not significantly associated with a decreased risk of incisional SSI compared to non-IOPL for patients with appendicitis (RR, 0.72 [95% CI, 0.18–2.86], *I*^*2*^ = 50%) (Fig. 3A). Incisional SSI occurred in five patients with peritonitis (17.2%) in the IOPL group and six patients with peritonitis (20.7%) in the non-IOPL group. The use of IOPL was not significantly associated with a decreased risk of incisional SSI compared to non-IOPL for patients with peritonitis (RR, 0.83 [95% CI, 0.29–2.43]) (Fig. 3A).

### Postoperative complication

Nine RCTs with 1096 patients reported on postoperative complication, seven included patients with appendicitis and two included patients with peritonitis [31, 32, 34–40]. Postoperative complication occurred in 52 patients with appendicitis (11.0%) in the IOPL group and 71 patients with appendicitis (13.2%) in the non-IOPL group. The use of IOPL was not significantly associated with a decreased risk of postoperative complication compared to non-IOPL for patients with appendicitis (RR, 0.74 [95% CI, 0.39–1.41], *I*^*2*^ = 64%) (Fig. 3B). Postoperative complication occurred in 12 patients with peritonitis (28.6%) in the IOPL group and 11 patients with peritonitis (25.6%) in the non-IOPL group. The use of IOPL was not significantly associated with a decreased risk of postoperative complication compared to non-IOPL for patients with peritonitis (RR, 1.11 [95% CI, 0.55–2.23], *I*^*2*^ = 0%) (Fig. 3B).

### Reoperation

**Fig. 4:**
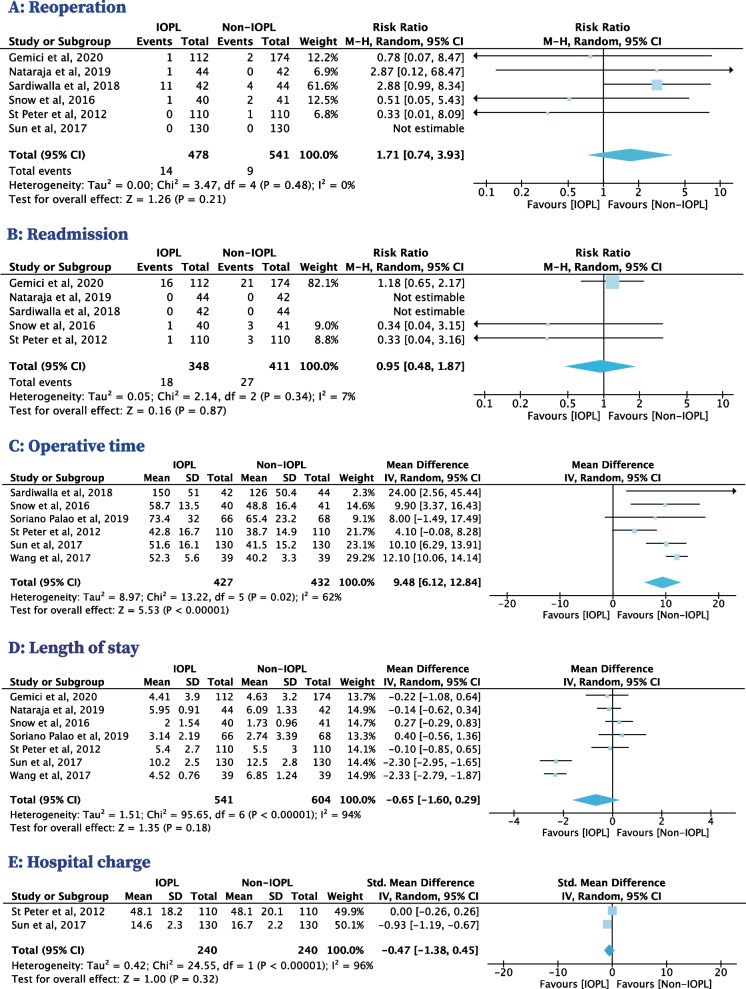
Secondary outcomes in patients with appendicitis who used IOPL compared with patients who did not

Six RCTs with 1019 patients reported on reoperation, in which all included patients were appendicitis [33–35, 37, 38, 40]. Reoperation occurred in 14 patients with appendicitis (2.9%) in the IOPL group and 9 patients with appendicitis (1.7%) in the non-IOPL group. The use of IOPL was not associated with a significantly decreased risk of reoperation compared with non-IOPL in patients with appendicitis (RR, 1.71[95% CI, 0.74–3.93], *I*^*2*^=0%) (Fig. 4A).

### Readmission

Five RCTs with 759 patients reported on readmission, in which all included patients were appendicitis [33, 34, 37, 38, 40]. Readmission occurred in 18 patients with appendicitis (5.2%) in the IOPL group and 27 patients with appendicitis (6.6%) in the non-IOPL group. The pooled estimates demonstrated that IOPL use was not associated with a significantly decreased risk of readmission compared with non-IOPL in patients with appendicitis (RR, 0.95 [95% CI, 0.48–1.87], *I*^*2*^ = 7%) (Fig. 4B).

### Operative time, LOS and hospital charge

Six RCTs reported on operative time [33–37, 39], seven RCTs reported on LOS [33–36, 38–40], and two RCTs reported on hospital charge [33, 35]. These studies only involve patients with appendicitis [33–40]. The results showed that compared with the no-IOPL group, the use of IOPL somewhat prolonged the operation time in patients with appendicitis (MD, 9.48 min [95% CI, 6.12–12.84], *I*^*2*^ = 62%). Results of LOS (MD = -0.65 days, 95% CI [-1.60 to 0.29], *I*^*2*^ = 94%) and hospital charge (SMD = -0.47, 95% CI [-1.38 to 0.45], *I*^*2*^ = 96%) were highly heterogenous (Fig. 4C–E). We found that different health systems were a source of high heterogeneity, and the heterogeneity of LOS (MD = 0.02 days, 95% CI [-0.27 to 0.31], *I*^*2*^ = 0%) and hospital charge (SMD = 0.00, 95% CI [-0.26 to 0.26]) was reduced after excluding studies of Sun et al. [35] and Wang et al. [36] from China.

### Sensitive analysis and publication bias

Sensitivity analysis for mortality was robust, while the result for IAA was less robust. The study of Sardiwalla et al. [37] had the greatest impact on the results of IAA. After excluding the study of Sardiwalla et al. [37], the RR of IAA changed from 1.05 (95% CI, 0.78–1.42) to 0.87 (95% CI, 0.62–1.24), without affecting the conclusion. A further analysis found that the study of Sardiwalla et al. [37] was stopped prematurely by the internal review due to the excess risk experienced by the IOPL group (Additional file 1: Fig. S3). The result of Egger's test for IAA showed that there was no significant evidence of publication bias (*P* = 0.72) (Additional file 1: Fig. S4).

### Quality of evidence

For patients with appendicitis, the certainty of evidence for mortality, IAA, incisional SSI, postoperative complication, reoperation, readmission, was downgraded from “high” to “moderate” by one level due to the wide confidence intervals of the findings. We did not find any possible downgraded factor for the outcomes of operative time, therefore, the certainty of the evidence for operative time is “high”. For patients with peritonitis, the certainty of evidence for mortality, IAA, incisional SSI, and postoperative complications was downgraded from “high” to “low” by two levels due to the small sample size and wide confidence intervals of the findings. (Additional file 1: Table S5).

## Discussion

This meta-analysis included ten RCTs with a total of 1318 patients, of which the majority concerned patients with appendicitis. We found that the use of IOPL did not provide additional benefits compared with non-IOPL with regards to mortality, IAA, incisional SSI, postoperative complication, reoperation, and readmission in patients with appendicitis. The benefits of the IOPL with saline for peritonitis patients are unclear due to the small sample size of the research. In the future, large, high-quality RCTs will be required to examine how IOPL affects individuals with peritonitis and other abdominal infections.

This SR included two studies on peritonitis and eight studies on appendicitis. Previous studies demonstrated that saline lavage reduced aerobic and anaerobic bacteria counts in peritoneal fluid, but it did not provide additional benefits for the outcomes of mortality and IAA [41–43]. The results of our SR are consistent with these previous findings except for reoperation [17–19]. A SR by Oweira et al. [21] reported that non-IOPL only during laparoscopic surgery for complicated appendicitis is associated with a lower reoperation rate (odds ratio [OR], 0.37 [95% CI, 0.14–0.96]) compared with peritoneal irrigation. However, we found that the two RCTs [33, 34] included by Oweira et al. [21] had problems with the extraction of reoperation data, which led to inconsistent findings with our review.

Many surgeons believe that “Dilution is the solution to pollution” [44]. However, moderate-quality evidence from our study does not support this view. The possible mechanisms for the ineffectiveness of IOPL were as follows [45, 46]: (1) bacteria adhere to the peritoneal mesothelial cells, such that irrigations cannot decrease the microorganism load on the peritoneum; (2) irrigation may cause bacterial dislocation and diffuse or remote inoculation, leading to pollution by spreading microorganisms; (3) irrigation may dilute mediators of phagocytosis such as opsonic proteins and immunoglobulins. In addition, high-quality evidence showed that IOPL with saline can prolong the operation time by about 10 min. Further, a retrospective study of 8168 patients with complicated appendicitis showed that every 1-min increase in operative time independently increased the likelihood of any SSI (OR, 1.010 [95% CI, 1.008–1.013]) and readmission (OR, 1.004 [95% CI, 1.000–1.007) [47]. The occurrence of SSI will not only increase the patient's hospital stay by 7 to 10 days, but also increase the cost of each readmission by 20,000 to 28,000 US dollars [48–50].

The 2017 World Society of Emergency Surgery guidelines [7] suggested that *Routine use of intraoperative irrigation for appendectomies does not prevent intra-abdominal abscess formation and may be avoided*, while the 2017 World Surgical Infection Society guideline [8] suggested, *Use of irrigation with crystalloid fluid to remove visible debris and gross contamination before abdominal closure in patients with IAI, generally limiting lavage to those areas with gross involvement as an adjunct to the source control procedure.* The main reason for the inconsistency of recommendations was that the guidelines did not use evidence from SRs when making their recommendations, but used the results of observational studies or RCTs. In 2005, a survey of the United Kingdom showed that 97% of surgeons used IOPL, and nearly half of them used saline for peritoneal irrigation [12]. However, current moderate-quality evidence does not support the routine use of IOPL in patient with appendicitis. Therefore, CPGs for IAI should consider updating the recommendations to avoid inappropriate use of IOPL, with the associated waste of time and medical resources.

This study has several limitations. First, most of the included studies on IAIs focused on appendicitis, while there are no studies that focus on other types of IAIs (e.g., pancreatitis, fecal peritonitis and etc.). Therefore, generalizing the results of this study to other types of IAIs may not be sufficient. Second, subgroup effects could not be evaluated when there were less than two trials in each subgroup. In addition, subgroup analyses were restricted by the study-level nature of the data. Most of the included articles did not clearly define the type of infection and population. Third, the Cochrane risk of bias tool used to assess the quality of surgical studies may have been relatively lenient, and other researchers may have different evaluation criteria.

## Conclusion

Evidence from moderate-quality studies suggested that the use of IOPL with saline was not associated with a reduced risk of mortality, IAA, incisional SSI, postoperative complication, reoperation, or readmission in patients with appendicitis when compared to non-IOPL. Therefore, the regular use of IOPL with saline in patients with appendicitis should be avoid. An investigation is still needed to determine the advantages of IOPL for IAI caused by other types of abdominal infections.

## Supplementary Information

**Additional file 1. Additional Table 1.** Search strategy; **Additional Table 2**. Definition for each outcome; **Additional Table 3**. Details on the missing SDs imputation for each outcome; **Additional Table 4**. Characteristics of excluded studies; **Additional Table 5**. GRADE assessment (summary of findings table); **Additional Figure 1**. Risk of bias assessment; **Additional Figure 2**. Forest plots with subgroup analysis of primary outcomes; **Additional Figure 3**. Sensitivity analysis of primary outcomes; **Additional Figure 4**. Publication bias (Egger’s test).

## Data Availability

All data analyzed during this study are included in this published article.

## Abbreviations

IOPL: Intraoperative peritoneal lavage
IAI: Intra-abdominal infection
RCT: Randomized controlled trial
GRADE: The Grading of Recommendations Assessment, Development and Evaluation
WSES: World Society of Emergency Surgery
WSIS: World Surgical Infection Society
IDSA: Infectious Diseases Society of America
CSS: Canadian Surgical Society
CSSIIC: Chinese Society of Surgical Infection and Intensive Care
CPG: Clinical practice guideline
IAA: Intra-abdominal abscess
SSI: Surgical site infection
RR: Risk ratio
HIC: High-income country
LMIC: Low- and middle-income country
CI: Confidence interval
MD: Mean difference
SMD: Standardized mean difference
PRISMA: Preferred reporting items for systematic reviews and meta-analysis
SR: Systematic review
