# Supplementary material for: Effectiveness of intraoperative peritoneal lavage with saline in patient with intra-abdominal infections: a systematic review and meta-analysis
Source: World J Emerg Surg. 2023 Mar 29;18:24. doi: 10.1186/s13017-023-00496-6 (PMC10061899; doi:10.1186/s13017-023-00496-6)
Supplement: Supplementary file 1 — Additional file 1. Additional Table 1. Search strategy; Additional Table 2. Definition for each outcome; Additional Table 3. Details on the missing SDs imputation for each outcome; Additional Table 4. Characteristics of excluded studies; Additional Table 5. GRADE assessment (summary of findings table); Additional Figure 1. Risk of bias assessment; Additional Figure 2. Forest plots with subgroup analysis of primary outcomes; Additional Figure 3. Sensitivity analysis of primary outcomes; Additional Figure 4. Publication bias (Egger’s test). [file 13017_2023_496_MOESM1_ESM.docx]

**Additional Table 1. Search strategy**

| **MEDLINE**   1. "Intraabdominal Infections"[MeSH] 2. “Intraabdominal Infection*”[Title/Abstract] 3. “Intra-abdominal infection*”[Title/Abstract] 4. “Intra-abdominal sepsis”[Title/Abstract] 5. “Intraabdominal sepsis”[Title/Abstract] 6. “Abdominal infection*”[Title/Abstract] 7. “abdomen infection*”[Title/Abstract] 8. “abdominal cavity infection*”[Title/Abstract] 9. “IAI” [Title/Abstract] 10. “peritoneal sepsis” [Title/Abstract] 11. “Intraperitoneal infection*” [Title/Abstract] 12. “peritoneal infection*” [Title/Abstract] 13. OR/#1-#12 14. "Suppurative appendicitis"[Title/Abstract] 15. "Purulent appendicitis"[Title/Abstract] 16. "Purulent ecphyaditis"[Title/Abstract] 17. "Suppurative ecphyaditis"[Title/Abstract] 18. "phlegmonous appendicitis"[Title/Abstract] 19. "Ruptured Appendicitis"[Title/Abstract] 20. "appendix rupture"[Title/Abstract] 21. "Perforated Appendicitis"[Title/Abstract] 22. "Perforated ecphyaditis"[Title/Abstract] 23. "appendicitis perforation"[Title/Abstract] 24. "appendicitis perforata"[Title/Abstract] 25. OR/#14-#24 26. "Peritonitis"[MeSH] 27. peritonitis[Title/Abstract] 28. perivisceritis[Title/Abstract] 29. OR/#26-#28 30. "Pyogenic cholecystitis"[Title/Abstract] 31. "purulent cholecystitis"[Title/Abstract] 32. "suppurative cholecystitis"[Title/Abstract] 33. "pyogenic cholangitis"[Title/Abstract] 34. "pyogeniccholangitis"[Title/Abstract] 35. "Suppurative cholangitis"[Title/Abstract] 36. "AOSC"[Title/Abstract] 37. OR/#30-#36 38. “Pancreatitis, Acute Necrotizing”[MeSH] 39. "severe acute pancreatitis"[Title/Abstract] 40. "acute severe pancreatitis"[Title/Abstract] 41. "Acute Necrotizing Pancreatitis"[Title/Abstract] 42. "serious acute pancreatitis"[Title/Abstract] 43. "SAP"[Title/Abstract] 44. OR/#38-#43 45. "Stomach Rupture"[MeSH] 46. "gastric perforation"[Title/Abstract] 47. "stomach perforation"[Title/Abstract] 48. "gastrobrosia"[Title/Abstract] 49. "perforation of stomach"[Title/Abstract] 50. "Stomach Rupture*"[Title/Abstract] 51. "Gastric Rupture*"[Title/Abstract] 52. OR/#45-#51 53. "Intestinal Perforation"[MeSH] 54. "Intestinal Perforation*"[Title/Abstract] 55. "intestine perforation"[Title/Abstract] 56. "gut perforation"[Title/Abstract] 57. "bowel perforation"[Title/Abstract] 58. "intestinal rupture"[Title/Abstract] 59. "enterorrhexis"[Title/Abstract] 60. "rupture of intestine"[Title/Abstract] 61. OR/#53-#60 62. "Intestinal Obstruction"[MeSH] 63. "Strangulation obstruction"[Title/Abstract] 64. "strangulated intestinal obstruction"[Title/Abstract] 65. "obstructing colon cancer"[Title/Abstract] 66. "obstructed colon cancer"[Title/Abstract] 67. "obstructing rectal cancer"[Title/Abstract] 68. "obstructed rectal cancer"[Title/Abstract] 69. "obstructing colorectal cancer"[Title/Abstract] 70. "obstructed colorectal cancer"[Title/Abstract] 71. OR/#62-#70 72. "Mesenteric Ischemia"[MeSH] 73. "Mesenteric Venous Thrombos*"[Title/Abstract] 74. "mesenteric ischaemia*"[Title/Abstract] 75. "mesentery ischemia"[Title/Abstract] 76. "mesentery ischaemia"[Title/Abstract] 77. OR/#72-#76 78. #13 OR #25 OR #29 OR #37 OR #44 OR #52 OR #61 OR #71 OR #77 79. "Therapeutic Irrigation"[MeSH] 80. lavage*[Title/Abstract] 81. irrigat*[Title/Abstract] 82. rins*[Title/Abstract] 83. wash[Title/Abstract] 84. douch*[Title/Abstract] 85. flush*[Title/Abstract] 86. syring*[Title/Abstract] 87. swash[Title/Abstract] 88. swill[Title/Abstract] 89. OR/#79-#88 90. "Surgical Procedures, Operative"[MeSH] 91. "General Surgery"[MeSH] 92. Operat*[Title/Abstract] 93. surger*[Title/Abstract] 94. surgical[Title/Abstract] 95. intraoperat*[Title/Abstract] 96. intra-operat*[Title/Abstract] 97. Peri-operat*[Title/Abstract] 98. Perioperat*[Title/Abstract] 99. OR/#90-#198 100. #78 AND #89 AND #99   **EMBASE**   1. 'abdominal infection'/exp 2. 'intraabdominal infection*':ti,ab 3. 'intra-abdominal infection*':ti,ab 4. 'intra-abdominal sepsis':ti,ab 5. 'intraabdominal sepsis':ti,ab 6. 'abdominal infection*':ti,ab 7. 'abdomen infection*':ti,ab 8. 'abdominal cavity infection*':ti,ab 9. 'iai':ti,ab 10. 'peritoneal sepsis':ti,ab 11. 'intraperitoneal infection*':ti,ab 12. 'peritoneal infection*':ti,ab 13. OR/#1-#12 14. 'purulent appendicitis':ti,ab 15. 'suppurative appendicitis':ti,ab 16. 'purulent ecphyaditis':ti,ab 17. 'suppurative ecphyaditis':ti,ab 18. 'phlegmonous appendicitis':ti,ab 19. 'ruptured appendicitis':ti,ab 20. 'appendix rupture':ti,ab 21. 'perforated appendicitis':ti,ab 22. 'perforated ecphyaditis':ti,ab 23. 'appendicitis perforation':ti,ab 24. 'appendicitis perforata':ti,ab 25. OR/#14-#24 26. 'peritonitis'/exp 27. 'peritonitis':ti,ab 28. 'perivisceritis':ti,ab 29. OR/#26-#28 30. 'pyogenic cholecystitis':ti,ab 31. 'purulent cholecystitis':ti,ab 32. 'suppurative cholecystitis':ti,ab 33. 'pyogenic cholangitis':ti,ab 34. 'pyogeniccholangitis':ti,ab 35. 'suppurative cholangitis':ti,ab 36. 'aosc':ti,ab 37. OR/#30-#36 38. 'severe acute pancreatitis'/exp 39. 'severe acute pancreatitis':ti,ab 40. 'acute severe pancreatitis':ti,ab 41. 'acute necrotizing pancreatitis':ti,ab 42. 'serious acute pancreatitis':ti,ab 43. 'sap':ti,ab 44. OR/#38-#43 45. 'stomach rupture'/exp 46. 'gastric perforation':ti,ab 47. 'stomach perforation':ti,ab 48. 'gastrobrosia':ti,ab 49. 'perforation of stomach':ti,ab 50. 'stomach rupture*':ti,ab 51. 'gastric rupture*':ti,ab 52. OR/#45-#51 53. 'intestine perforation'/exp 54. 'intestinal perforation*':ti,ab 55. 'intestine perforation*':ti,ab 56. 'gut perforation*':ti,ab 57. 'bowel perforation*':ti,ab 58. 'intestinal rupture*':ti,ab 59. 'enterorrhexis':ti,ab 60. 'rupture of intestine':ti,ab 61. OR/#53-#60 62. 'intestine obstruction'/exp 63. 'strangulation obstruction':ti,ab 64. 'strangulated intestinal obstruction':ti,ab 65. 'obstructing rectal cancer':ti,ab 66. 'obstructed rectal cancer':ti,ab 67. 'obstructing colon cancer':ti,ab 68. 'obstructed colon cancer':ti,ab 69. 'obstructing colorectal cancer':ti,ab 70. 'obstructed colorectal cancer':ti,ab 71. OR/#62-#70 72. 'mesenteric ischemia'/exp 73. 'mesenteric venous thrombos*':ti,ab 74. 'mesentery ischemia':ti,ab 75. 'mesenteric ischaemia*':ti,ab 76. 'mesentery ischaemia':ti,ab 77. OR/#72-#76 78. #13 OR #25 OR #29 OR #37 OR #44 OR #52 OR #61 OR #71 OR #77 79. 'lavage'/exp 80. wash*:ti,ab 81. irrigat*:ti,ab 82. rins*:ti,ab 83. douch*:ti,ab 84. lavage*:ti,ab 85. flush*:ti,ab 86. syring*:ti,ab 87. swash:ti,ab 88. swill:ti,ab 89. OR/#79-#88 90. 'surgery'/exp 91. 'general surgery'/exp 92. 'operat*':ti,ab 93. 'surger*':ti,ab 94. 'surgical':ti,ab 95. 'intraoperat*':ti,ab 96. 'intra-operat*':ti,ab 97. 'peri-operat*':ti,ab 98. 'perioperat*':ti,ab 99. OR/#90-#98 100. #78 AND #89 AND #99 101. #100[medline]/lim 102. #100 NOT #101   **Web of Science**   1. TOPIC: (“Intraabdominal Infection*”) 2. TOPIC: (“Intra-abdominal infection*”) 3. TOPIC: (“Intra-abdominal sepsis”) 4. TOPIC: (“Intraabdominal sepsis”) 5. TOPIC: (“Abdominal infection*”) 6. TOPIC: (“abdomen infection*”) 7. TOPIC: (“abdominal cavity infection*”) 8. TOPIC: (“IAI”) 9. TOPIC: (“peritoneal sepsis”) 10. TOPIC: (“Intraperitoneal infection*”) 11. TOPIC: (“peritoneal infection*”) 12. OR/#1-#11 13. TOPIC: (peritonitis) 14. TOPIC: (perivisceritis) 15. #13 OR #14 16. TOPIC: ("Suppurative appendicitis") 17. TOPIC: ("Purulent appendicitis") 18. TOPIC: ("Purulent ecphyaditis") 19. TOPIC: ("Suppurative ecphyaditis") 20. TOPIC: ("phlegmonous appendicitis") 21. TOPIC: ("Ruptured Appendicitis") 22. TOPIC: ("appendix rupture") 23. TOPIC: ("Perforated Appendicitis") 24. TOPIC: ("Perforated ecphyaditis") 25. TOPIC: ("appendicitis perforation") 26. TOPIC: ("appendicitis perforata") 27. OR/#16-#26 28. TOPIC: ("Pyogenic cholecystitis") 29. TOPIC: ("purulent cholecystitis") 30. TOPIC: ("suppurative cholecystitis") 31. TOPIC: ("pyogenic cholangitis") 32. TOPIC: ("pyogeniccholangitis") 33. TOPIC: ("Ruptured Appendicitis") 34. TOPIC: ("Suppurative cholangitis") 35. TOPIC: ("AOSC") 36. OR/#28-#35 37. TOPIC: ("severe acute pancreatitis") 38. TOPIC: ("acute severe pancreatitis") 39. TOPIC: ("Acute Necrotizing Pancreatitis") 40. TOPIC: ("serious acute pancreatitis") 41. TOPIC: ("SAP") 42. OR/#37-#41 43. TOPIC: ("gastric perforation") 44. TOPIC: ("stomach perforation") 45. TOPIC: ("gastrobrosia") 46. TOPIC: ("perforation of stomach") 47. TOPIC: ("Stomach Rupture*") 48. TOPIC: ("Gastric Rupture*") 49. TOPIC: ("Intestinal Perforation*") 50. TOPIC: ("intestine perforation*") 51. TOPIC: ("gut perforation*") 52. TOPIC: ("bowel perforation*") 53. TOPIC: ("intestinal rupture*") 54. TOPIC: ("rupture of intestine") 55. TOPIC: ("enterorrhexis") 56. OR/#43-#55 57. TOPIC: ("obstructing colorectal cancer") 58. TOPIC: ("obstructed colorectal cancer") 59. TOPIC: ("obstructing rectal cancer") 60. TOPIC: ("obstructed rectal cancer") 61. TOPIC: ("obstructing colorectal cancer") 62. TOPIC: ("obstructed colorectal cancer ") 63. TOPIC: ("Strangulation obstruction") 64. TOPIC: ("strangulated intestinal obstruction") 65. OR/#57-#64 66. TOPIC: ("Mesenteric Venous Thrombos*") 67. TOPIC: ("mesentery ischemia") 68. TOPIC: ("mesentery ischaemia") 69. TOPIC: ("mesenteric ischaemia*") 70. OR/#66-#69 71. #12 OR #15 OR #27 OR #36 OR #42 OR #56 OR #65 OR #70 72. TOPIC: ("Operat*") 73. TOPIC: ("surger*") 74. TOPIC: ("surgical") 75. TOPIC: ("intraoperat*") 76. TOPIC: ("intra-operat*") 77. TOPIC: ("Peri-operat*") 78. TOPIC: ("Perioperat*") 79. OR/#72-#78 80. TOPIC: (lavage*) 81. TOPIC: (irrigat*) 82. TOPIC: (wash*) 83. TOPIC: (rins*) 84. TOPIC: (douch*) 85. TOPIC: (flush*) 86. TOPIC: (syring*) 87. TOPIC: (swash) 88. TOPIC: (swill) 89. OR/#80-#88 90. #71 AND #79 AND #89   **Cochrane Library**   1. MeSH descriptor: [Intraabdominal Infections] explode all trees 2. (“Intraabdominal Infection*”):ti,ab,kw 3. (“Intra-abdominal infection*”):ti,ab,kw 4. (“Intra-abdominal sepsis”):ti,ab,kw 5. (“Intraabdominal sepsis”):ti,ab,kw 6. (“Intra-abdominal infection*”):ti,ab,kw 7. (“abdomen infection*”):ti,ab,kw 8. (“abdominal cavity infection*”):ti,ab,kw 9. (“IAI”):ti,ab,kw 10. (“peritoneal sepsis”):ti,ab,kw 11. (“Intraperitoneal infection*”):ti,ab,kw 12. (“peritoneal infection*”):ti,ab,kw 13. OR/#1-#12 14. ("Suppurative appendicitis"):ti,ab,kw 15. ("Purulent appendicitis"):ti,ab,kw 16. ("Purulent ecphyaditis"):ti,ab,kw 17. ("Suppurative ecphyaditis"):ti,ab,kw 18. ("phlegmonous appendicitis"):ti,ab,kw 19. ("Ruptured Appendicitis"):ti,ab,kw 20. ("appendix rupture"):ti,ab,kw 21. ("Perforated Appendicitis"):ti,ab,kw 22. ("Perforated ecphyaditis"):ti,ab,kw 23. ("appendicitis perforation"):ti,ab,kw 24. ("appendicitis perforata"):ti,ab,kw 25. OR/#14-#24 26. MeSH descriptor: [Peritonitis] explode all trees 27. (peritonitis):ti,ab,kw 28. (perivisceritis):ti,ab,kw 29. OR/#26-#28 30. ("Pyogenic cholecystitis"):ti,ab,kw 31. ("purulent cholecystitis"):ti,ab,kw 32. ("suppurative cholecystitis"):ti,ab,kw 33. ("pyogenic cholangitis"):ti,ab,kw 34. ("pyogeniccholangitis"):ti,ab,kw 35. ("Suppurative cholangitis"):ti,ab,kw 36. (AOSC):ti,ab,kw 37. OR/#30-#36 38. MeSH descriptor: [Pancreatitis, Acute Necrotizing] explode all trees 39. ("severe acute pancreatitis"):ti,ab,kw 40. ("acute severe pancreatitis"):ti,ab,kw 41. ("Acute Necrotizing Pancreatitis"):ti,ab,kw 42. ("serious acute pancreatitis"):ti,ab,kw 43. (SAP):ti,ab,kw 44. OR/#38-#43 45. MeSH descriptor: [Stomach Rupture] explode all trees 46. ("gastric perforation"):ti,ab,kw 47. ("stomach perforation"):ti,ab,kw 48. ("gastrobrosia"):ti,ab,kw 49. ("perforation of stomach"):ti,ab,kw 50. ("Stomach Rupture*"):ti,ab,kw 51. ("Gastric Rupture*"):ti,ab,kw 52. OR/#45-#51 53. MeSH descriptor: [Intestinal Perforation] explode all trees 54. ("Intestinal Perforation*"):ti,ab,kw 55. ("intestine perforation*"):ti,ab,kw 56. ("gut perforation*"):ti,ab,kw 57. ("bowel perforation*"):ti,ab,kw 58. ("intestinal rupture*"):ti,ab,kw 59. ("rupture of intestine"):ti,ab,kw 60. ("enterorrhexis"):ti,ab,kw 61. OR/#53-#60 62. MeSH descriptor: [Intestinal Obstruction] explode all trees 63. ("Strangulation obstruction"):ti,ab,kw 64. ("strangulated intestinal obstruction"):ti,ab,kw 65. ("obstructing colon cancer"):ti,ab,kw 66. ("obstructed colon cancer"):ti,ab,kw 67. ("obstructing rectal cancer"):ti,ab,kw 68. ("obstructed rectal cancer"):ti,ab,kw 69. ("obstructing colorectal cancer"):ti,ab,kw 70. ("obstructed colorectal cancer"):ti,ab,kw 71. OR/#62-#70 72. MeSH descriptor: [Mesenteric Ischemia] explode all trees 73. ("Mesenteric Venous Thrombos*"):ti,ab,kw 74. ("mesentery ischemia"):ti,ab,kw 75. ("mesentery ischaemia"):ti,ab,kw 76. ("mesenteric ischaemia*"):ti,ab,kw 77. OR/#72-#76 78. #13 OR #25 OR #29 OR #37 OR #44 OR #52 OR #61 OR #71 OR #77 79. MeSH descriptor: [Therapeutic Irrigation] explode all trees 80. (lavage*):ti,ab,kw 81. (irrigat*):ti,ab,kw 82. (wash*):ti,ab,kw 83. (rins*):ti,ab,kw 84. (douch*):ti,ab,kw 85. (flush*):ti,ab,kw 86. (syring*):ti,ab,kw 87. (swash):ti,ab,kw 88. (swill):ti,ab,kw 89. OR/#79-#88 90. MeSH descriptor: [General Surgery] explode all trees 91. MeSH descriptor: [Surgical Procedures, Operative] explode all trees 92. ("Operat*"):ti,ab,kw 93. ("surger*"):ti,ab,kw 94. (surgical):ti,ab,kw 95. (intraoperat*):ti,ab,kw 96. (intra-operat*):ti,ab,kw 97. ("Peri-operat*"):ti,ab,kw 98. ("Perioperat*"):ti,ab,kw 99. OR/#90-#98 100. #78 AND #89 AND #99   **CBM**   1. "腹腔灌洗" [不加权:扩展] 2. "清洗"[常用字段:智能] 3. "冲洗" [常用字段:智能] 4. "灌洗" [常用字段:智能] 5. OR/#1-#4 6. "腹腔感染"[常用字段:智能] 7. "腹部感染"[常用字段:智能] 8. "腹膜炎"[常用字段:智能] 9. "化脓性阑尾炎"[常用字段:智能] 10. "阑尾破裂"[常用字段:智能] 11. "穿孔性阑尾炎"[常用字段:智能] 12. "化脓性胆囊炎"[常用字段:智能] 13. "化脓性胆管炎"[常用字段:智能] 14. "胰腺炎, 急性坏死性"[不加权:扩展] 15. "急性重症胰腺炎"[常用字段:智能] 16. "胃穿孔"[常用字段:智能] 17. "胃破裂"[不加权:扩展] 18. "胃破裂"[常用字段:智能] 19. "肠穿孔"[不加权:扩展] 20. "肠穿孔"[常用字段:智能] 21. "肠破裂"[常用字段:智能] 22. "结直肠癌梗阻"[常用字段:智能] 23. "结肠癌梗阻"[常用字段:智能] 24. "直肠癌梗阻"[常用字段:智能] 25. "梗阻性结肠癌"[常用字段:智能] 26. "梗阻性直肠癌"[常用字段:智能] 27. "梗阻性结直肠癌"[常用字段:智能] 28. "绞窄性肠梗阻"[常用字段:智能] 29. "肠系膜血栓"[常用字段:智能] 30. OR/#6-#29 31. "手术期间"[不加权:扩展] 32. "外科手术"[不加权:扩展] 33. "手术"[常用字段:智能] 34. "外科"[常用字段:智能] 35. "术中"[常用字段:智能] 36. "普外"[常用字段:智能] 37. OR/#31-#36 38. #5 AND #30 AND #37   **Wanfang**   1. 主题:("清洗") 2. 主题:("冲洗") 3. 主题:("灌洗") 4. OR/#1-#3 5. 主题:("腹腔感染") 6. 主题:("腹部感染") 7. 主题:("腹膜炎") 8. 主题:("化脓性阑尾炎") 9. 主题:("阑尾破裂") 10. 主题:("穿孔性阑尾炎") 11. 主题:("化脓性胆囊炎") 12. 主题:("化脓性胆管炎") 13. 主题:("急性重症胰腺炎") 14. 主题:("胃穿孔") 15. 主题:("胃破裂") 16. 主题:("肠穿孔") 17. 主题:("肠破裂") 18. 主题:("结直肠癌梗阻") 19. 主题:("结肠癌梗阻") 20. 主题:("肠系膜血栓") 21. 主题:("梗阻性结直肠癌") 22. 主题:("绞窄性肠梗阻") 23. OR/#5-#22 24. 主题:("手术") 25. 主题:("外科") 26. 主题:("术中") 27. 主题:("普外") 28. OR/#24-#27 29. #4 AND #23 AND #28   **CNKI**   1. 主题:("清洗") 2. 主题:("冲洗") 3. 主题:("灌洗") 4. OR/#1-#3 5. 主题:("腹腔感染") 6. 主题:("腹部感染") 7. 主题:("腹膜炎") 8. 主题:("化脓性阑尾炎") 9. 主题:("阑尾破裂") 10. 主题:("穿孔性阑尾炎") 11. 主题:("化脓性胆囊炎") 12. 主题:("化脓性胆管炎") 13. 主题:("急性重症胰腺炎") 14. 主题:("胃穿孔") 15. 主题:("胃破裂") 16. 主题:("肠穿孔") 17. 主题:("肠破裂") 18. 主题:("结直肠癌梗阻") 19. 主题:("结肠癌梗阻") 20. 主题:("肠系膜血栓") 21. 主题:("梗阻性结直肠癌") 22. 主题:("绞窄性肠梗阻") 23. OR/#5-#22 24. 主题:("手术") 25. 主题:("外科") 26. 主题:("术中") 27. 主题:("普外") 28. OR/#24-#27 29. #4 AND #23 AND #28   **World Health Organization** **International Clinical Trials Registry Platform (**<http://www.who.int/ictrp/en/>**)**  **(**peritoneal OR Abdominal OR peritoneum OR appendicitis) AND (wash* OR irrigation* OR rins* OR douch* OR lavage* OR flush*)  **ClinicalTrials.gov (** <https://clinicaltrials.gov/>**)**  (Condition or disease: Abdominal infection OR appendicitis) AND (Intervention/treatment: Irrigation)  **Google Scholar**  (Peritoneal Lavage OR irrigation) AND (Abdominal infection OR appendicitis) |
| --- |

**Additional Table 2. Definition for each outcome**

| **2.1 Mortality:** Mortality were defined as within 30 days after operation.  **2.2 Intra-abdominal abscess (IAA):** An intra-abdominal abscess is a collection of pus or infected fluid that is surrounded by inflamed tissue inside the belly. It can involve any abdominal organ, or it can settle in the folds of the bowel. IAA was defined in the study as a fluid collection that developed postoperatively and confirmed by computerized tomography imaging and by the radiologist’s final interpretation of the image.  **2.3 Incisional Surgical Site Infection:** Incisional SSI was defined as the combination of superficial and deep incisional SSI occurring within 30 days after surgery, conforming to the CDC guidelines^1^.  **2.4 Postoperative complication:** It conforms to the definition of National Surgical Quality Improvement Program (NSQIP)^2^. It not only includes infectious complications such as abscess rate and wound infection, but non-infectious complications such as bowel obstruction also will be included.  **2.5 Reoperation:** An operation to correct a condition not corrected by a previous operation or to correct the complications of a previous operation.  **2.6 Readmission:** It was defined that the patient readmits to hospital within 30 days of the principal surgical procedure for any reason.  **2.7** **Operative time:** From surgery start time to surgery end time in minutes.  **2.8 Length of hospital stay:** Inpatient days are calculated by subtracting day of admission from day of discharge.  **2.9 Hospital charge:** Total medical costs for the primary hospital stay within 30 postoperative days.  **2.10 References**  1. Berríos-Torres SI, Umscheid CA, Bratzler DW, et al. Centers for Disease Control and Prevention Guideline for the Prevention of Surgical Site Infection, 2017. JAMA Surg. 2017;152(8):784-791. doi:10.1001/jamasurg.2017.0904  2. American College of Surgery. Userguide for the ACS NSQIP participant use data file. 2018. https://www.facs.org/quality-programs/acs-nsqip/participant-use. |
| --- |

**Additional Table 3. Details on the missing SDs imputation for each outcome**

| *Methods for imputation of missing standard deviations*  We used published standard deviations (SDs), where available. When standard errors instead of SDs were presented, the former was converted to SDs.^1^ If both were missing, we estimated SDs from P values or confidence interval (CIs) according to the recommendations of the Cochrane Handbook for Systematic Reviews.^1^ We also estimated SDs from graphs when they were missing in tables or in text. If studies reported median, range and/or interquartile range, we used median to impute the missing mean and calculated SDs.^2^ If none of these options are viable, we imputed the missing SDs using pooled SDs from other studies included in our systematic review following the formula below:^3^  ${SD}_{pooled}=\sqrt{\frac{\sum\left( n_{i}-1 \right){SD}_{I}^{2}}{\sum(n_{i}-1)}}$  *Operative time*  Only one study (Snow et al, 2016) did not report the mean and SDs in their report. The study (Snow et al, 2016) reported median and inter-quartile range (IQR) between experimental and control groups. We imputed the mean and standard deviation from the sample size, median, IQR.^2^  *Length of stay*  Two studies (Snow et al, 2016; Nataraja et al, 2019) did not report the mean and SDs in their report. Two studies (Snow et al, 2016; Nataraja et al, 2019) reported median (IQR or range) between experimental and control groups. We imputed the mean and SD from the sample size, median, range and/or IQR.^2^  *References*  1. Deeks JJ, Higgins JPT, Altman DG (editors). Chapter 10: Analysing data and undertaking meta-analyses. In: Higgins JPT, Thomas J, Chandler J, Cumpston M, Li T, Page MJ, Welch VA (editors). Cochrane Handbook for Systematic Reviews of Interventions version 6.3 (updated February 2022). Cochrane, 2022. Available from www.training.cochrane.org/handbook.  2. Wan X, Wang W, Liu J, et al. Estimating the sample mean and standard deviation from the sample size, median, range and/or interquartile range. BMC Med Res Methodol. 2014 19; 14:135. |
| --- |

**Additional Table 4. Characteristics of excluded studies**

| **No.** | **Study** | **Reason for exclusion** |
| --- | --- | --- |
| 1 | Lee TG, Nam S, Lee HS, Lee JH, Hong YK, Kang JG. Irrigation Versus Suction Alone During Laparoscopic Appendectomy for Uncomplicated Acute Appendicitis. Ann Coloproctol. 2020;36(1):30-34. doi:10.3393/ac.2019.06.25 | Observational study |
| 2 | Ohno Y, Furui J, Kanematsu T. Treatment strategy when using intraoperative peritoneal lavage for perforated appendicitis in children: a preliminary report. Pediatr Surg Int. 2004;20(7):534-537. doi:10.1007/s00383-004-1210-y | Observational study |
| 3 | Hartwich JE, Carter RF, Wolfe L, et al. The effects of irrigation on outcomes in cases of perforated appendicitis in children. J Surg Res. 2013;180(2):222-225. doi:10.1016/j.jss.2012.04.043 | Observational study |
| 4 | Cho J, Park I, Lee D, Sung K, Baek J, Lee J. Risk Factors for Postoperative Intra-Abdominal Abscess after Laparoscopic Appendectomy: Analysis for Consecutive 1,817 Experiences. Dig Surg. 2015;32(5):375-381. doi:10.1159/000438707 | Observational study |
| 5 | Akkoyun I, Tuna AT. Advantages of abandoning abdominal cavity irrigation and drainage in operations performed on children with perforated appendicitis. J Pediatr Surg. 2012;47(10):1886-1890. doi:10.1016/j.jpedsurg.2012.03.049 | Observational study |
| 6 | Moore CB, Smith RS, Herbertson R, Toevs C. Does use of intraoperative irrigation with open or laparoscopic appendectomy reduce post-operative intra-abdominal abscess?. Am Surg. 2011;77(1):78-80. | Observational study |
| 7 | Toki A, Ogura K, Horimi T, et al. Peritoneal lavage versus drainage for perforated appendicitis in children. Surg Today 1995;25:207–10. | Observational study |
| 8 | Escolino M, Becmeur F, Saxena A, et al. Infectious Complications After Laparoscopic Appendectomy in Pediatric Patients with Perforated Appendicitis: Is There a Difference in the Outcome Using Irrigation and Suction Versus Suction Only? Results of a Multicentric International Retrospective Study. *J Laparoendosc Adv Surg Tech A*. 2018;28(10):1266-1270. doi:10.1089/lap.2018.0061 | Observational study |
| 9 | Hussain A, Mahmood H, Nicholls J, El-Hasani S. Prevention of intra-abdominal abscess following laparoscopic appendicectomy for perforated appendicitis: a prospective study. Int J Surg. 2008;6(5):374-377. doi:10.1016/j.ijsu.2008.06.006 | Observational study |
| 10 | Viney R, Isaacs C, Chelmow D. Intra-abdominal irrigation at cesarean delivery: a randomized controlled trial. Obstet Gynecol. 2012;119(6):1106-1111. doi:10.1097/AOG.0b013e3182460d09 | Patient without intra-abdominal infection |
| 11 | Sargin MA, Yassa M, Turunc M, Karadogan FO, Aydin S, Tug N. Abdominal irrigation during cesarean section: is it beneficial for the control of postoperative pain and gastrointestinal disturbance? A randomized controlled, double-blind trial. Int J Clin Exp. 2016;9(2):3416-24. doi:IJCEM0018432 | Patient without intra-abdominal infection |
| 12 | O'Boyle CJ, deBeaux AC, Watson DI, et al. Helium vs carbon dioxide gas insufflation with or without saline lavage during laparoscopy. Surg Endosc. 2002;16(4):620-625. doi:10.1007/s00464-001-8218-3 | Patient without intra-abdominal infection |
| 13 | Cho OY, Yoon HS. Effect of the exchange of saline used in surgical procedures on surgical site infection. Taehan Kanho Hakhoe Chi. 2004;34(3):467-476. doi:10.4040/jkan.2004.34.3.467 | Patient without intra-abdominal infection |
| 14 | Güngördük K, Asicioglu O, Celikkol O, Ark C, Tekırdağ AI. Does saline irrigation reduce the wound infection in caesarean delivery?. J Obstet Gynaecol. 2010;30(7):662-666. doi:10.3109/01443615.2010.494206 | Patient without intra-abdominal infection |
| 15 | Temizkan O, Asıcıoglu O, Güngördük K, Asıcıoglu B, Yalcin P, Ayhan I. The effect of peritoneal cavity saline irrigation at cesarean delivery on maternal morbidity and gastrointestinal system outcomes. J Matern Fetal Neonatal Med. 2016;29(4):651-655. doi:10.3109/14767058.2015.1015415 | Patient without intra-abdominal infection |
| 16 | Tanaka K, Matsuo K, Kawaguchi D, et al. Randomized clinical trial of peritoneal lavage for preventing surgical site infection in elective liver surgery. J Hepatobiliary Pancreat Sci. 2015;22(6):446-453. doi:10.1002/jhbp.222 | Patient without intra-abdominal infection |
| 17 | St Peter SD, Shah SR, Adibe OO, et al. Saline vs Tissue Plasminogen Activator Irrigations after Drain Placement for Appendicitis-Associated Abscess: A Prospective Randomized Trial. J Am Coll Surg. 2015;221(2):390-396. doi:10.1016/j.jamcollsurg.2015.03.043 | Irrigation with antibiotic |
| 18 | Sindelar WF, Brower ST, Merkel AB, Takesue EI. Randomised trial of intraperitoneal irrigation with low molecular weight povidone-iodine solution to reduce intra-abdominal infectious complications. J Hosp Infect. 1985;6 Suppl A:103-114. doi:10.1016/s0195-6701(85)80054-2 | Irrigation with antibiotic |
| 19 | Sherman JO, Luck SR, Borger JA. Irrigation of the peritoneal cavity for appendicitis in children: a double-blind study. J Pediatr Surg. 1976;11(3):371-374. doi:10.1016/s0022-3468(76)80191-1 | Irrigation with antibiotic |
| 20 | Raeeszadeh M, Hosseini SM, Khanmohammadi MT, Manoochehry S, Rasouli HR. Comparison of peritoneal lavage with normal saline and normal saline plus antibiotic in acute peritonitis. Trauma Monthly. 2017;22(5):4. | Irrigation with antibiotic |
| 21 | Anderson KT, Putnam LR, Bartz-Kurycki MA, et al. Povidone-iodine Irrigation for Pediatric Perforated Appendicitis May Be Protective: A Bayesian Pilot Randomized Controlled Trial. Ann Surg. 2020;271(5):827-833. doi:10.1097/SLA.0000000000003398 | Irrigation with antibiotic |
| 22 | Nomikos IN, Katsouyanni K, Papaioannou AN. Washing with or without chloramphenicol in the treatment of peritonitis: a prospective, clinical trial. Surgery. 1986;99(1):20-25. | Irrigation with antibiotic |
| 23 | de Jong TE, Vierhout RJ, van Vroonhoven TJ. Povidone-iodine irrigation of the subcutaneous tissue to prevent surgical wound infections. Surg Gynecol Obstet. 1982;155(2):221-224. | Irrigation with antibiotic |
| 24 | NCT. Dilute Povidone-iodine Irrigation vs No Irrigation for Children With Acute, Perforated Appendicitis. 2016. https://clinicaltrials.gov/show/nct02664220 | Irrigation with antibiotic |
| 25 | Vennix S, Musters GD, Mulder IM, et al. Laparoscopic peritoneal lavage or sigmoidectomy for perforated diverticulitis with purulent peritonitis: a multicentre, parallel-group, randomised, open-label trial. Lancet. 2015;386(10000):1269-1277. doi:10.1016/S0140-6736(15)61168-0 | The control group did not meet the criteria |
| 26 | Kohl A, Rosenberg J, Bock D, et al. Two-year results of the randomized clinical trial DILALA comparing laparoscopic lavage with resection as treatment for perforated diverticulitis. Br J Surg. 2018;105(9):1128-1134. doi:10.1002/bjs.10839 | The control group did not meet the criteria |
| 27 | Thornell A, Angenete E, Bisgaard T, et al. Laparoscopic Lavage for Perforated Diverticulitis With Purulent Peritonitis: A Randomized Trial. Ann Intern Med. 2016;164(3):137-145. doi:10.7326/M15-1210 | The control group did not meet the criteria |
| 28 | Ponzano C, Hüscher CG, Overi D. Laparoscopic Lavage Is Feasible and Safe for the Treatment of Perforated Diverticulitis With Purulent Peritonitis: The First Results From the Randomized Controlled Trial DILALA. Ann Surg. 2017;265(5):e66-e67. doi:10.1097/SLA.0000000000001258 | The control group did not meet the criteria |
| 29 | Azhar N, Johanssen A, Sundström T, et al. Laparoscopic Lavage vs Primary Resection for Acute Perforated Diverticulitis: Long-term Outcomes From the Scandinavian Diverticulitis (SCANDIV) Randomized Clinical Trial. JAMA Surg. 2021;156(2):121-127. doi:10.1001/jamasurg.2020.5618 | The control group did not meet the criteria |
| 30 | Schwarz A, Bölke E, Peiper M, et al. Inflammatory peritoneal reaction after perforated appendicitis: continuous peritoneal lavage versus non lavage. Eur J Med Res. 2007;12(5):200-205. | Continuous post-operative lavage |
| 31 | Buanes TA, Andersen GP, Jacobsen U, Nygaard K. Perforated appendicitis with generalized peritonitis. Prospective, randomized evaluation of closed postoperative peritoneal lavage. Eur J Surg. 1991;157(4):277-279. | Continuous post-operative lavage |
| 32 | Hallerbäck B, Andersson C, Englund N, et al. A prospective randomized study of continuous peritoneal lavage postoperatively in the treatment of purulent peritonitis. Surg Gynecol Obstet. 1986;163(5):433-436. | Continuous post-operative lavage |
| 33 | Collin Y, Mayer S, Mosimann F. Irrigation versus suction alone during laparoscopic appendectomy for perforated appendicitis: a prospective randomized trial. Ann Surg. 2015;261(4):e98. doi:10.1097/SLA.0000000000000448 | Letter |
| 34 | Vohra RS. Irrigation versus suction alone during laparoscopic appendectomy for perforated appendicitis: a prospective randomized trial. Ann Surg. 2015;261(4):e111. doi:10.1097/SLA.0000000000000488 | Letter |

**Additional Table 5. GRADE assessment (summary of findings table)**

| **Certainty assessment** | | | | | | | **Summary of findings** | | |
| --- | --- | --- | --- | --- | --- | --- | --- | --- | --- |
| **Participants (studies)** | **Risk of bias** | **Inconsistency** | **Indirectness** | **Imprecision** | **Publication bias** | **Certainty of evidence** | **Study event rates (%)** | | **Effect size (95% CI)** |
|  |  |  |  |  |  |  | **With [non-IOPL]** | **With [IOPL]** |  |
| **Mortality (appendicitis)** | | | | | | | | | |
| 286 (1 RCT) | Not serious^a^ | Not serious | Not serious | Serious^c^ | None | ⨁⨁⨁◯ Moderate | 2/174  (1.1%) | 0/112  (0%) | **RR 0.31** (0.02 to 6.39) |
| **Mortality (peritonitis)** | | | | | | | | | |
| 87 (2 RCTs) | Not serious^a^ | Not serious | Not serious | Serious^d^ | None | ⨁⨁◯◯ Low | 10/43  (23.3%) | 10/44  (22.7%) | **RR 0.97** (0.45 to 2.09) |
| **Intra-abdominal abscess (appendicitis)** | | | | | | | | | |
| 1153 (7 RCTs) | Not serious^a^ | Not serious | Not serious | Serious^c^ | None | ⨁⨁⨁◯ Moderate | 72/609  (11.8%) | 67/544  (12.3%) | **RR 1.02** (0.70 to 1.48) |
| **Intra-abdominal abscess (peritonitis)** | | | | | | | | | |
| 79 (2 RCTs) | Not serious^a^ | Not serious | Not serious | Serious^d^ | None | ⨁⨁◯◯ Low | 2/39  (5.1%) | 2/40  (5.0%) | **RR 1.05** (0.16 to 6.98) |
| **Incisional surgical site infection (appendicitis)** | | | | | | | | | |
| 791 ( 5 RCTs) | Not serious^a^ | Not serious | Not serious | Serious^c^ | None | ⨁⨁⨁◯ Moderate | 16/426  (3.8%) | 12/365  (3.3%) | **RR 0.72** (0.18 to 2.86) |
| **Incisional surgical site infection (peritonitis)** | | | | | | | | | |
| 58 ( 1 RCT) | Not serious^a^ | Not serious | Not serious | Serious^d^ | None | ⨁⨁◯◯ Low | 5/29  (17.2%) | 6/29  (20.7%) | **RR 0.83** (0.29 to 2.43) |
| **Postoperative complication (appendicitis)** | | | | | | | | | |
| 1011 (7 RCTs) | Not serious^a^ | Not serious | Not serious | Serious^c^ | None | ⨁⨁⨁◯ Moderate | 71/538  (13.2%) | 52/473  (11.0%) | **RR 0.74** (0.39 to 1.41) |
| **Postoperative complication (peritonitis)** | | | | | | | | | |
| 85 (2 RCTs) | Not serious^a^ | Not serious | Not serious | Serious^d^ | None | ⨁⨁◯◯ Low | 11/43  (25.6%) | 12/42  (28.6%) | **RR 1.11** (0.55 to 2.32) |
| **Reoperation (appendicitis)** | | | | | | | | | |
| 1019 (6 RCTs) | Not serious^a^ | Not serious | Not serious | Serious^c^ | None | ⨁⨁⨁◯ Moderate | 9/541  (1.7%) | 14/478  (2.9%) | **RR 1.71** (0.74 to 3.93) |
| **Readmission (appendicitis)** | | | | | | | | | |
| 759 (5 RCTs) | Not serious^a^ | Not serious | Not serious | Serious^c^ | None | ⨁⨁⨁◯ Moderate | 27/411  (6.6%) | 18/348 (5.2%) | **RR 0.95** (0.48 to 1.87) |
| **Operative time (appendicitis)** | | | | | | | | | |
| 859 (6 RCTs) | Not serious^a^ | Not serious | Not serious | Not serious | None | ⨁⨁⨁⨁ High | NA/432  (NA) | NA/427  (NA) | **MD 9.48 min**  (6.12 to 12.84) |
| **Length of hospital stay (appendicitis)** | | | | | | | | | |
| 1203 (7 RCTs) | Not serious^a^ | Serious^b^ | Not serious | Serious^e^ | None | ⨁⨁◯◯ Low | NA/604  (NA) | NA/541  (NA) | **MD -0.65 days**  (-1.60 to 0.29) |
| **Hospital charge (appendicitis)** | | | | | | | | | |
| 480 (2 RCTs) | Not serious^a^ | Serious^b^ | Not serious | Serious^e^ | None | ⨁⨁◯◯ Low | NA/240  (NA) | NA/240  (NA) | **SMD -0.47**  (-1.38 to 0.45) |

**CI:** confidence interval; **MD:** mean difference; **RR:** risk ratio; **SMD:** standardised mean difference; NA: not applicable

**Explanation:**

a. Although the blindness of 10 RCTs was missing or unclear, it had little effect on the results of mortality, intra-abdominal abscess and other outcomes. Therefore, we did not consider downgrades in risk of bias;

b. Downgraded one level due to serious inconsistency because of statistical heterogeneity(*I^2^*> 75%);

c. Downgraded one level due to the wide confidence interval of results.

d. Downgraded two levels due to the sample size is lower than the optimal information size (OIS) and wide confidence interval of results.

e. Downgraded one level due to the sample size is lower than the optimal information size (OIS).

**Additional Figure 1. Risk of bias assessment**


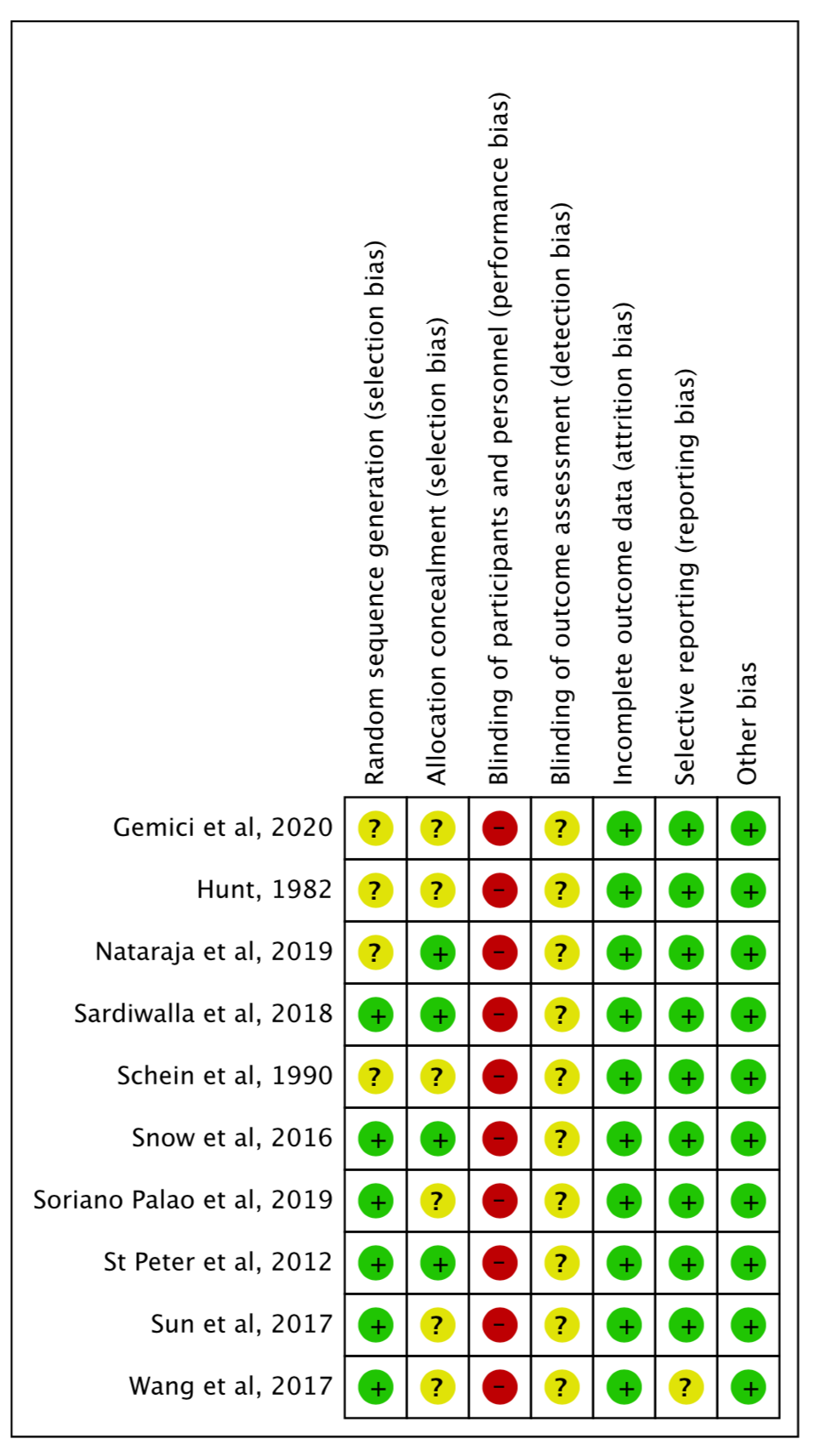


Figure S1.1 Risk of bias summary


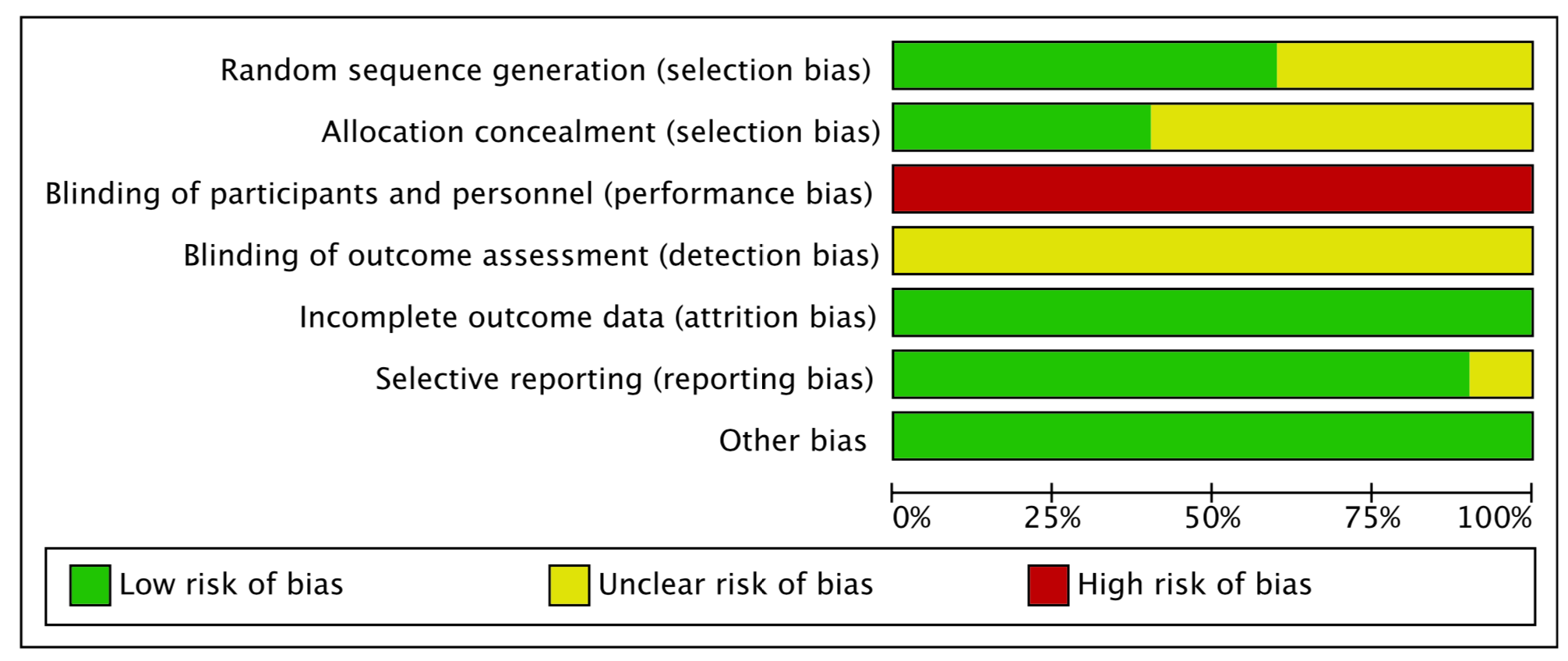


Figure S1.2 Risk of bias graph

**Additional Figure 2. Forest plots with subgroup analysis of primary outcomes**

**
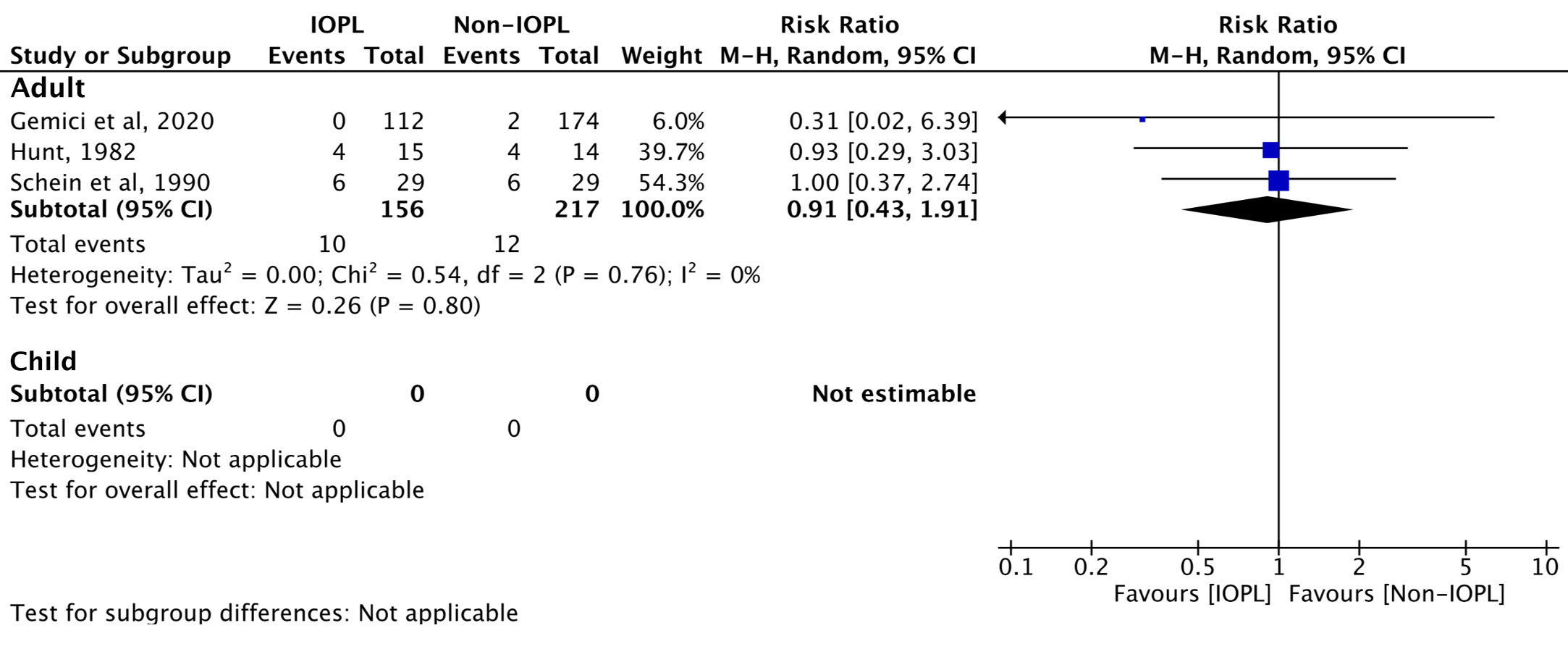
**

Figure S2.1 Subgroup analysis of mortality (Type of population)

**
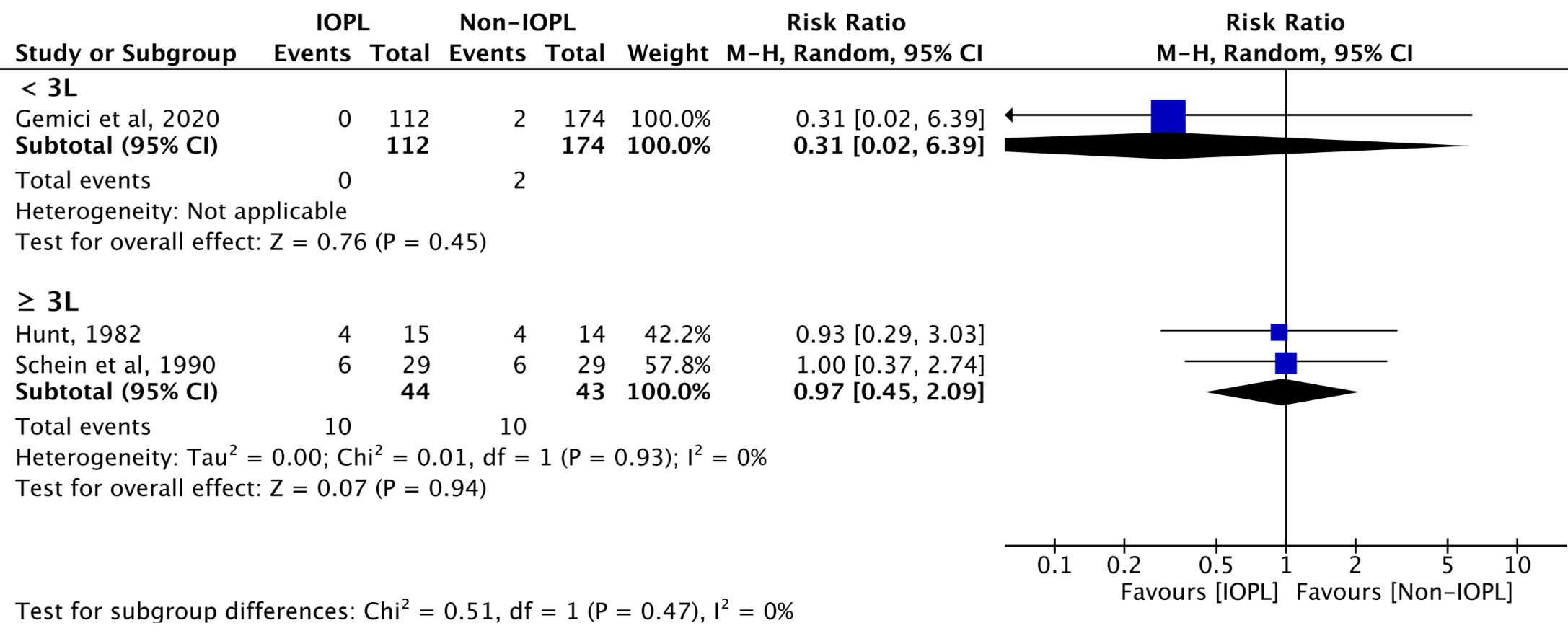
**

Figure S2.2 Subgroup analysis of mortality (Irrigation volume)

**
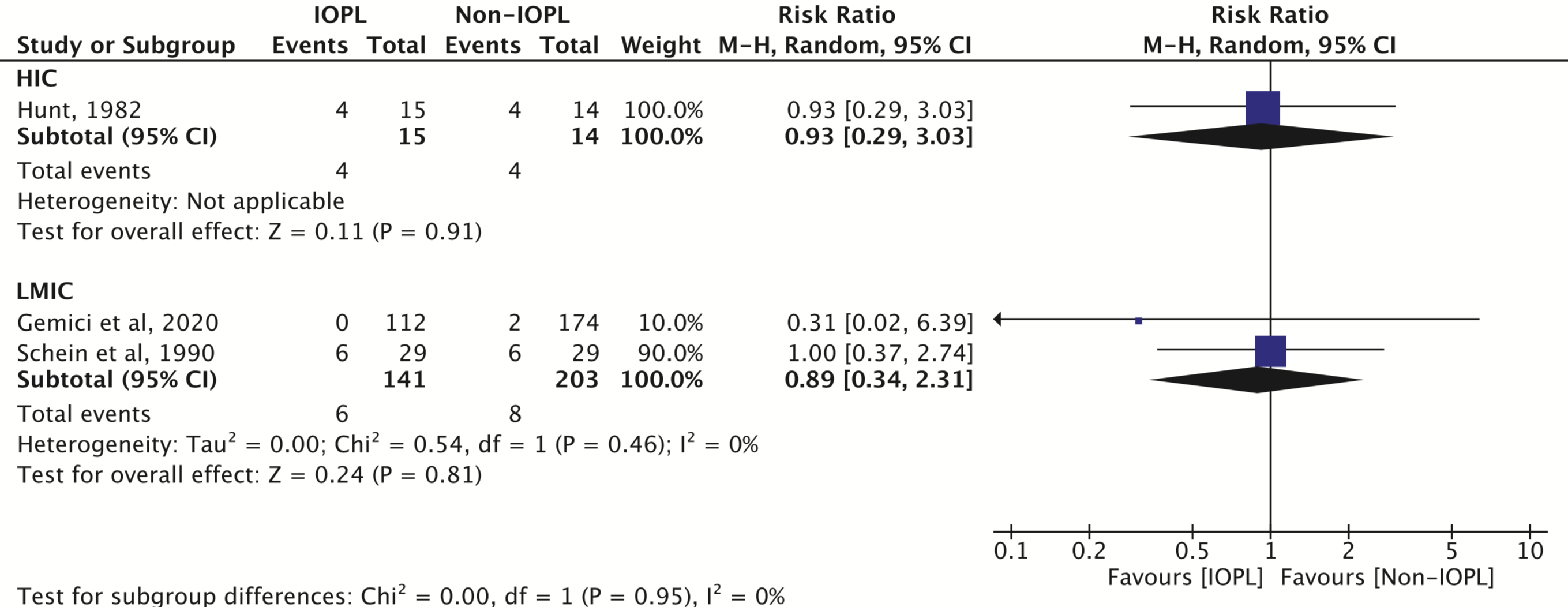
**

Figure S2.3 Subgroup analysis of mortality (Country income level)

**
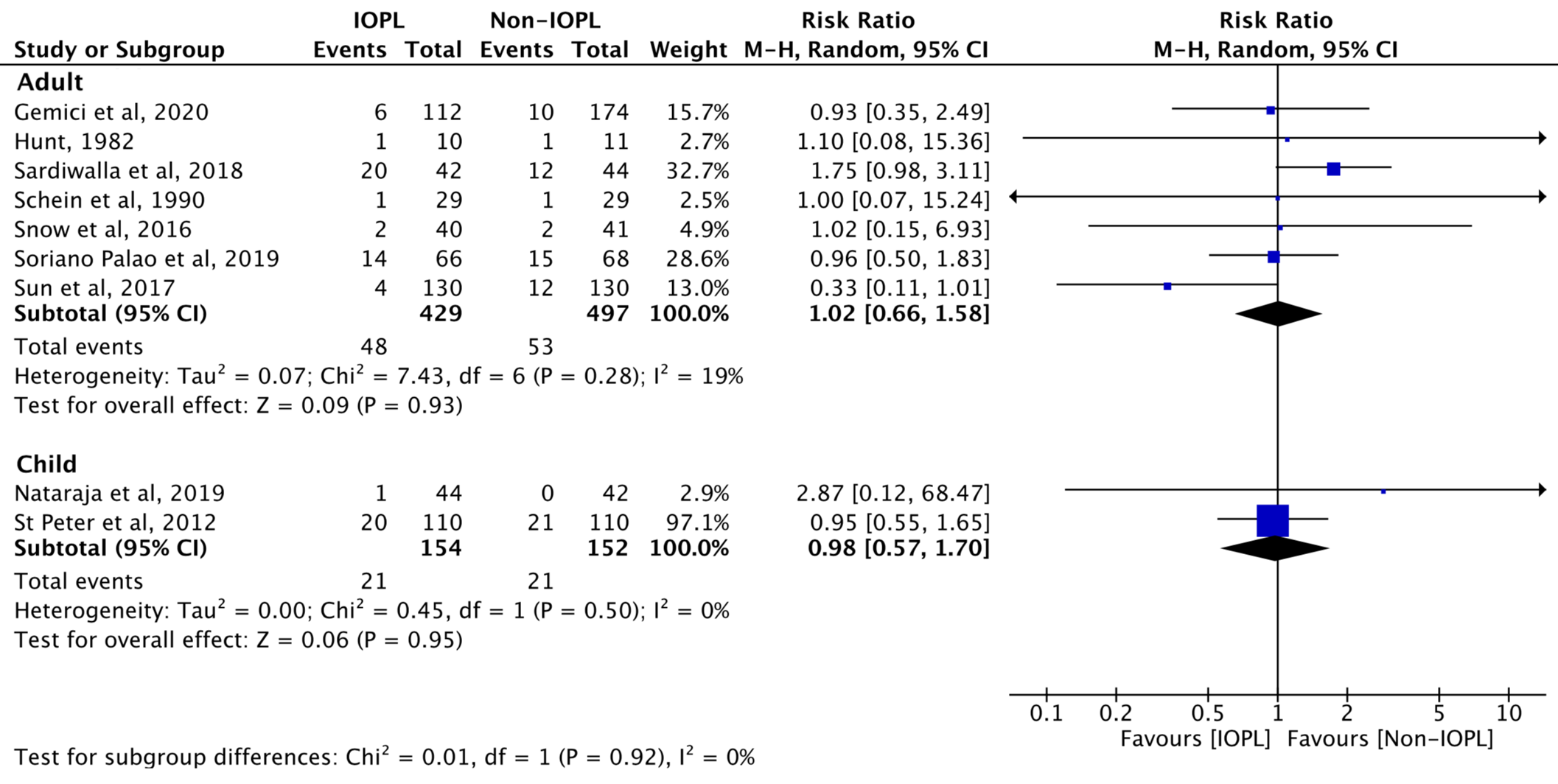
**

Figure S2.4 Subgroup analysis of intra-abdominal abscess (Type of population)

**
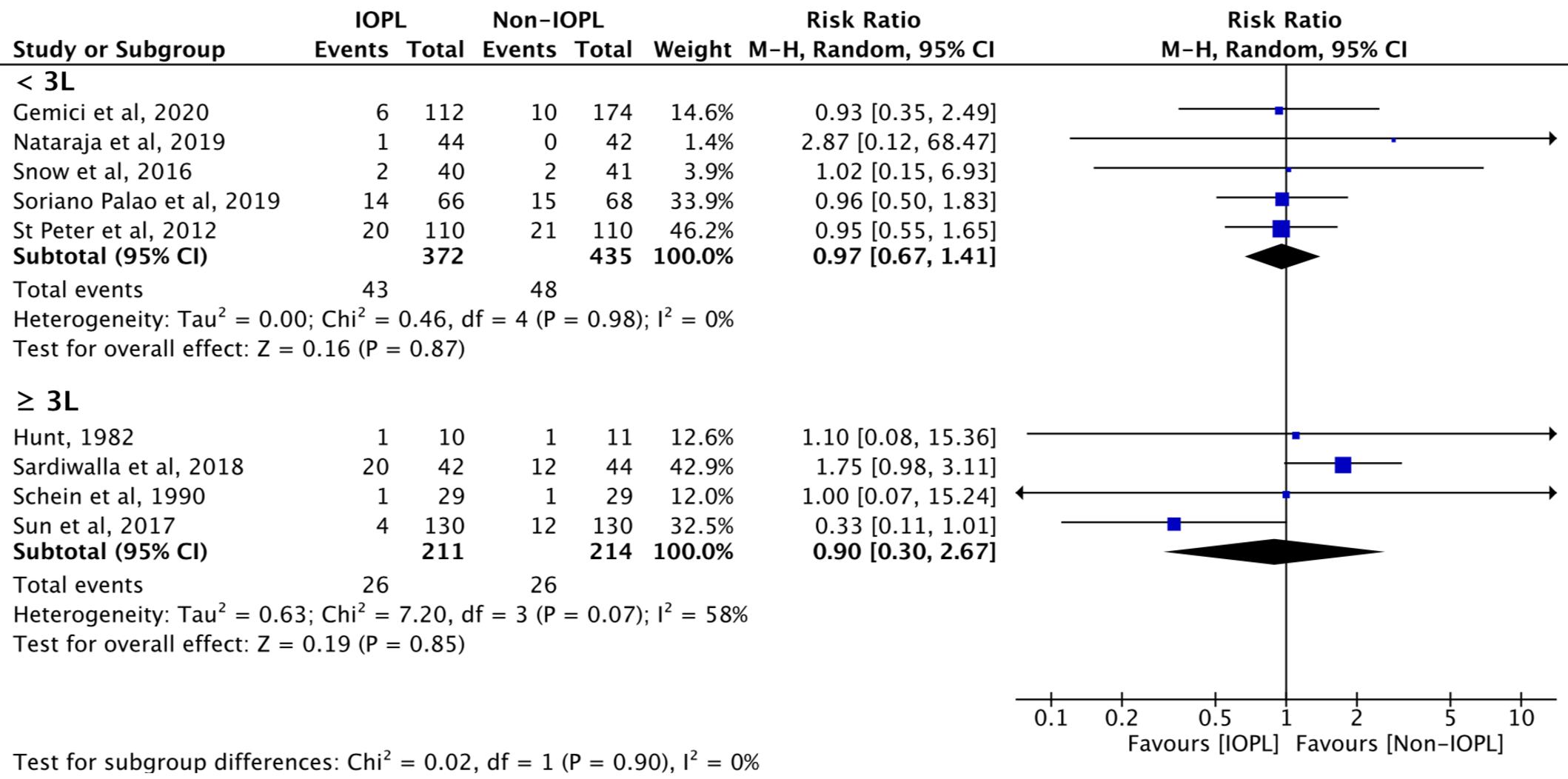
**

Figure S2.5 Subgroup analysis of intra-abdominal abscess (Irrigation volume)

**
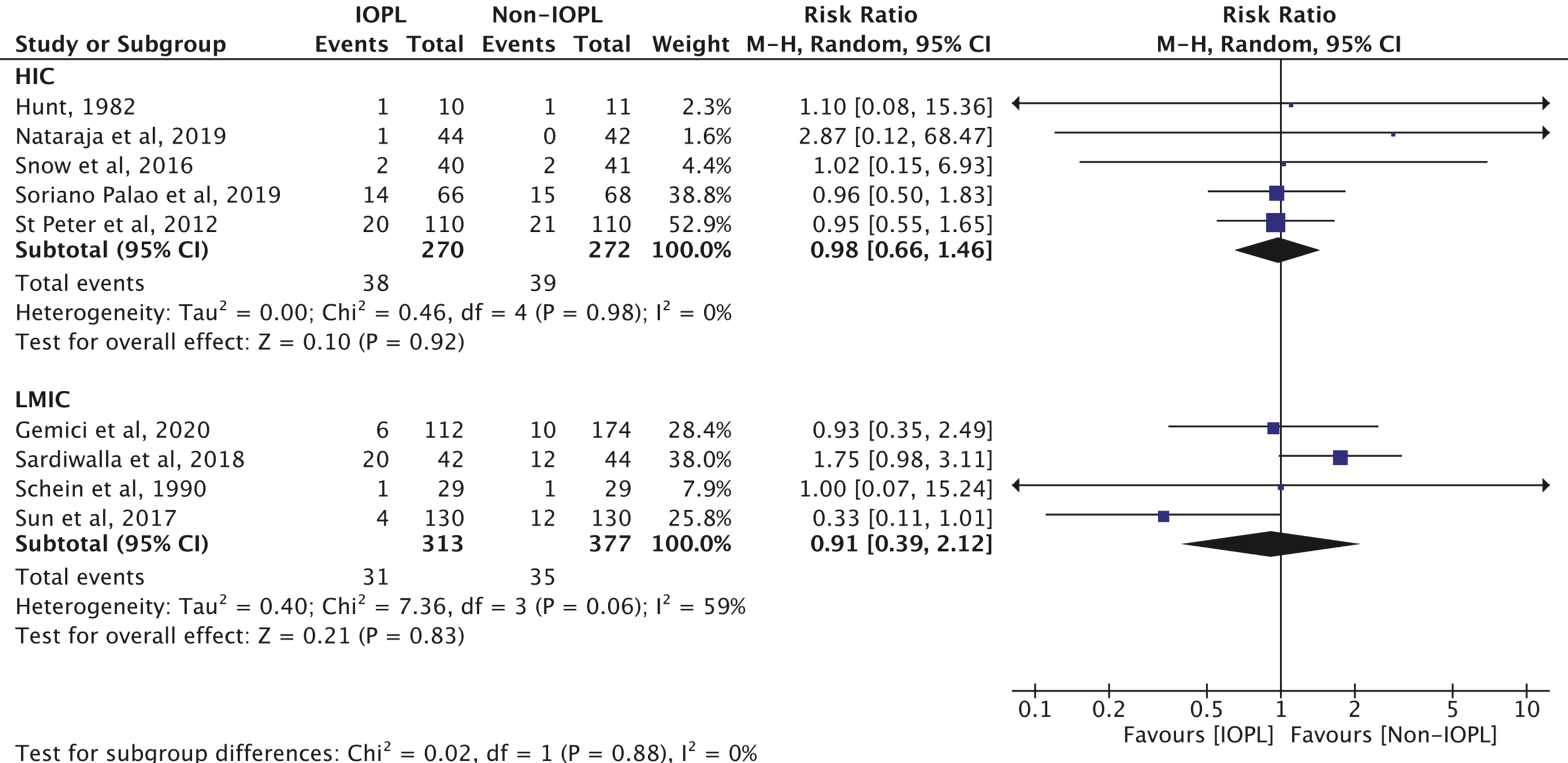
**

Figure S2.6 Subgroup analysis of intra-abdominal abscess (Country income level)

**Additional Figure 3. Sensitivity analysis of primary outcomes**

Sensitivity Analysis of Mortality

------------------------------------------------------------------------------------------------------------------

Study omitted | Estimate [95% Conf. Interval]

-------------------+---------------------------------------------------------------------------------------------

Gemici et al, 2020. | 0.97129613 0.45149419 2.0895422

Hunt, 1982 | 0.8896448 0.34195009 2.3145711

Schein et al, 1990 | 0.80710924 0.26901707 2.4215019

-------------------+---------------------------------------------------------------------------------------------

Combined | 0.90672677 0.43146406 1.9054969

-----------------------------------------------------------------------------------------------------------------


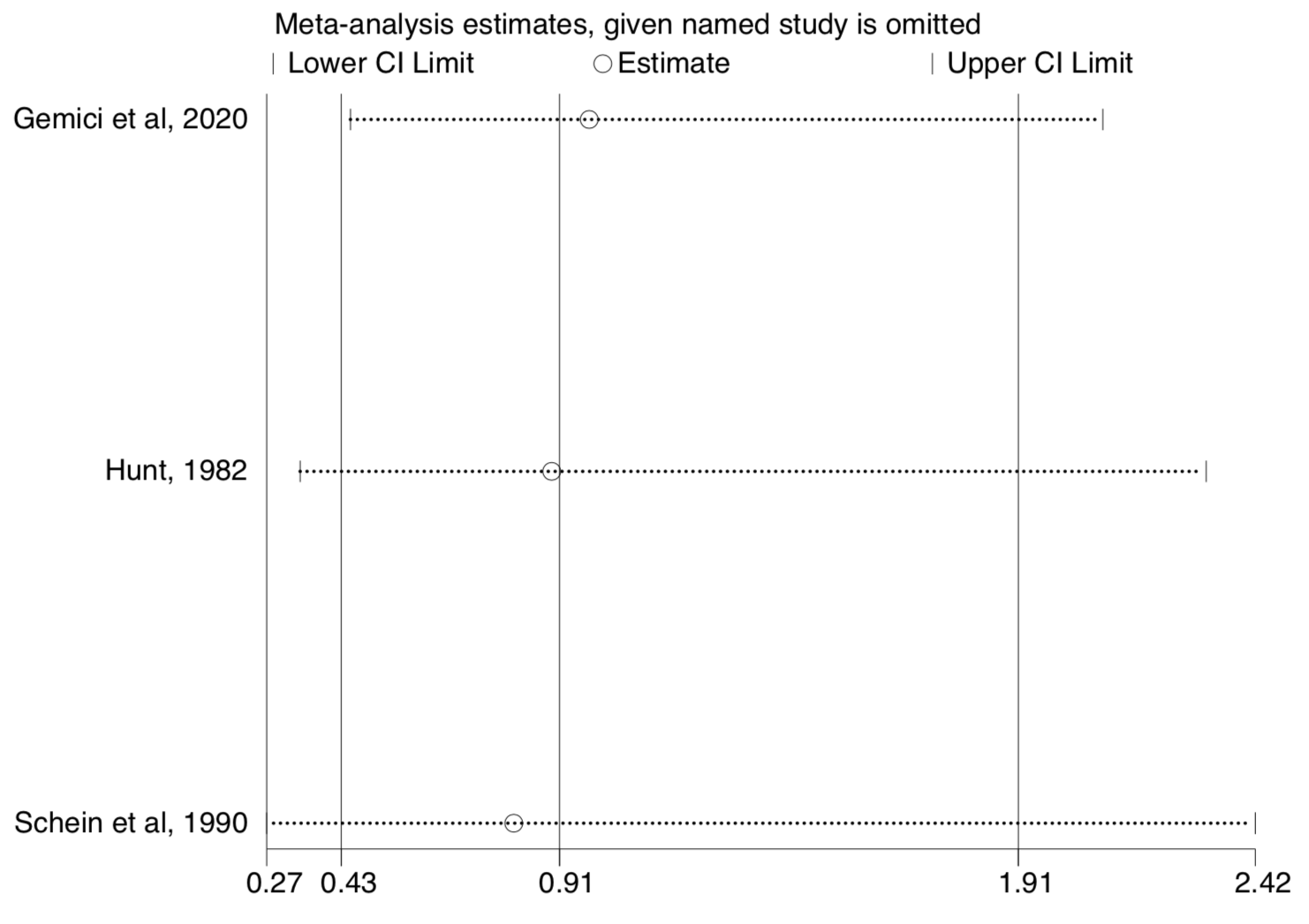


Figure S4.1 Sensitivity Analysis of Mortality

Sensitivity Analysis of Intra-abdominal Abscess

-------------------------------------------------------------------------------------------------------------

Study omitted | Estimate [95% Conf. Interval]

-------------------+-----------------------------------------------------------------------------------------

Gemici et al, 2020. | 1.0468104 0.73447442 1.4919674

Nataraja et al, 2019 | 1.0309501 0.74754852 1.4217913

Palao et al, 2019 | 1.0510927 0.71496433 1.5452464

Sardiwalla et al, 2018 | 0.87414825 0.61629784 1.2398796

Hunt, 1982 | 1.0340688 0.73886293 1.4472214

Schein et al, 1990 | 1.0356462 0.7401675 1.4490813

Snow et al, 2016 | 1.0350759 0.73731941 1.4530773

St Peter et al, 2012 | 1.061664 0.71198082 1.583091

Sun et al, 2017 | 1.1523585 0.84469312 1.5720861

-------------------+----------------------------------------------------------------------------------------

Combined | 1.0523345 .78036162 1.4190958

------------------------------------------------------------------------------------------------------------


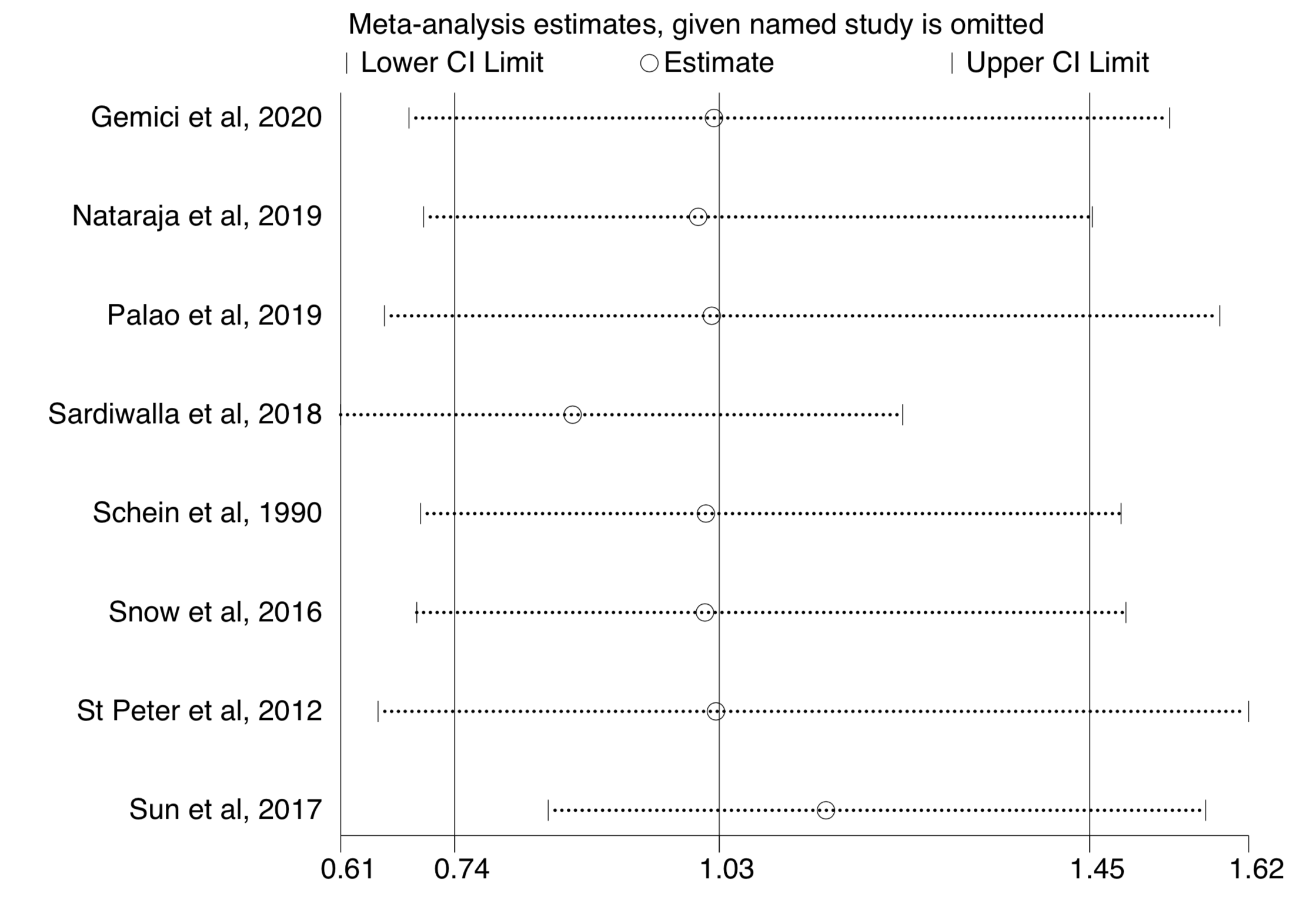


Figure S4.2 Sensitivity Analysis of Intra-abdominal Abscess

**Additional Figure 4. Publication bias (Egger’s test)**

Egger's test for small-study effects:

Regress standard normal deviate of intervention

effect estimates against its standard error

Number of studies = 9 Root MSE = 1.042


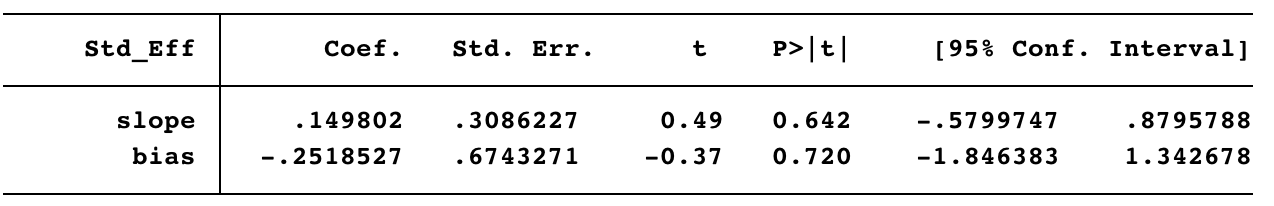


Test of H0: no small-study effects ***P* = 0.720**


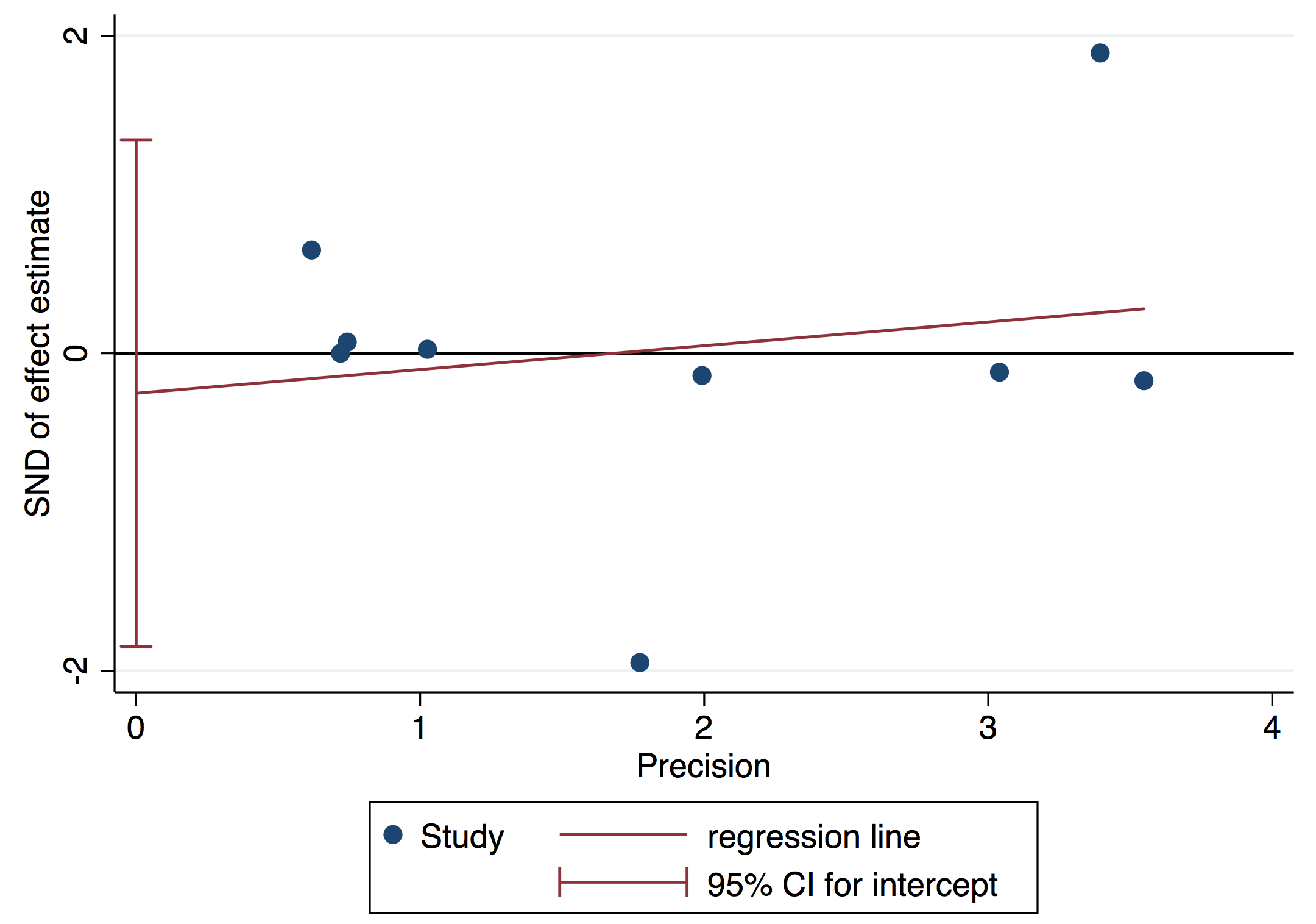


Egger’s test for Intra-abdominal Abscess
